# Kidney Angiotensin in Cardiovascular Disease: Formation and Drug Targeting

**DOI:** 10.1124/pharmrev.120.000236

**Published:** 2022-07

**Authors:** Hui Lin, Frank Geurts, Luise Hassler, Daniel Batlle, Katrina M. Mirabito Colafella, Kate M. Denton, Jia L. Zhuo, Xiao C. Li, Nirupama Ramkumar, Masahiro Koizumi, Taiji Matsusaka, Akira Nishiyama, Martin J. Hoogduijn, Ewout J. Hoorn, A.H. Jan Danser

**Affiliations:** Division of Pharmacology and Vascular Medicine (H.L., A.H.J.D.) and Division of Nephrology and Transplantation (F.G., M.J.H., E.J.H.), Department of Internal Medicine, Erasmus Medical Centre, Rotterdam, The Netherlands; Northwestern University Feinberg School of Medicine, Chicago, Illinois (L.H., D.B.); Monash University, Melbourne, Australia (K.M.M.C., K.M.D.); Tulane University School of Medicine, New Orleans, Louisiana (J.L.Z., X.C.L.); Division of Nephrology and Hypertension, University of Utah School of Medicine, Salt Lake City, Utah (N.R.); Division of Nephrology, Endocrinology, and Metabolism (M.K.) and Institute of Medical Sciences and Department of Basic Medicine (M.K., T.M.), Tokai University School of Medicine, Isehara, Japan; and Department of Pharmacology, Faculty of Medicine, Kagawa University, Miki-cho, Kita-gun, Japan (A.N.)

## Abstract

The concept of local formation of angiotensin II in the kidney has changed over the last 10–15 years. Local synthesis of angiotensinogen in the proximal tubule has been proposed, combined with prorenin synthesis in the collecting duct. Binding of prorenin via the so-called (pro)renin receptor has been introduced, as well as megalin-mediated uptake of filtered plasma-derived renin-angiotensin system (RAS) components. Moreover, angiotensin metabolites other than angiotensin II [notably angiotensin-(1-7)] exist, and angiotensins exert their effects via three different receptors, of which angiotensin II type 2 and Mas receptors are considered renoprotective, possibly in a sex-specific manner, whereas angiotensin II type 1 (AT_1_) receptors are believed to be deleterious. Additionally, internalized angiotensin II may stimulate intracellular receptors. Angiotensin-converting enzyme 2 (ACE2) not only generates angiotensin-(1-7) but also acts as coronavirus receptor. Multiple, if not all, cardiovascular diseases involve the kidney RAS, with renal AT_1_ receptors often being claimed to exert a crucial role. Urinary RAS component levels, depending on filtration, reabsorption, and local release, are believed to reflect renal RAS activity. Finally, both existing drugs (RAS inhibitors, cyclooxygenase inhibitors) and novel drugs (angiotensin receptor/neprilysin inhibitors, sodium-glucose cotransporter-2 inhibitors, soluble ACE2) affect renal angiotensin formation, thereby displaying cardiovascular efficacy. Particular in the case of the latter three, an important question is to what degree they induce renoprotection (e.g., in a renal RAS-dependent manner). This review provides a unifying view, explaining not only how kidney angiotensin formation occurs and how it is affected by drugs but also why drugs are renoprotective when altering the renal RAS.

**Significance Statement:**

Angiotensin formation in the kidney is widely accepted but little understood, and multiple, often contrasting concepts have been put forward over the last two decades. This paper offers a unifying view, simultaneously explaining how existing and novel drugs exert renoprotection by interfering with kidney angiotensin formation.

## I. Introduction

Angiotensin (Ang) II exerts multiple effects in the kidney (Kobori et al., 2007b), and it is now widely accepted that this involves locally synthesized rather than circulating Ang II. The beneficial effects of renin-angiotensin system (RAS) blockers in the kidney, often occurring independently of their blood pressure–lowering effects, support this view. Yet, how exactly this independent angiotensin generation occurs remains incompletely understood. For many years, it was thought to depend on angiotensinogen (AGT) synthesis in the kidney, combined with local renin synthesis in the collecting duct (CD). The discovery of a receptor for prorenin, the so-called (pro)renin receptor [(P)RR] (Nguyen et al., 2002), caused further excitement, as this might explain why the CD predominantly generated prorenin (i.e., renin’s inactive precursor). By binding to this receptor, prorenin would be able to display activity, and it even seemed to act as an agonist of this receptor. Hence, the (P)RR was welcomed as a new RAS member. Other members received wide attention as well, in particular the previously less studied Ang II type 2 (AT_2_) and Mas receptor. The latter recognizes Ang-(1-7). Remarkably, these two receptors are now believed to oppose the classic, deleterious effects of Ang II in the kidney, mediated via its type 1 (AT_1_) receptor. To our knowledge, this has not yet led to novel drugs for cardiovascular or kidney disease.

The presence of Ang-(1-7) in the kidney requires an enzyme capable of generating this metabolite in sufficient quantities. Here angiotensin-converting enzyme 2 (ACE2) comes into play. Given its additional role as coronavirus receptor, the relationship between the RAS, its inhibitors, and coronavirus disease 2019 (COVID-19) is currently hotly debated (Danser et al., 2020) and novel treatment options like soluble ACE2 are likely to emerge. Angiotensin receptor/neprilysin inhibition (ARNI) and sodium-glucose cotransporter-2 (SGLT2) inhibition, originally introduced for the treatment of heart failure and diabetes respectively, turned out to be renoprotective as well, possibly by affecting the renal RAS. This is also true for another group of drugs, the cyclooxygenase (COX) inhibitors, and novel drugs capable of suppressing AGT generation. The latter, by acting in a liver-specific manner, have helped to address the origin of tissue AGT and have also altered our view on urinary AGT as a biomarker (Roksnoer et al., 2016a).

This review provides an up-to-date review of the renal RAS, focusing primarily on how it allows/facilitates local angiotensin generation in health and disease. We start by evaluating the various concepts of renin synthesis in the kidney at its classic location in the juxtaglomerular (JG) cells versus the CD, simultaneously considering the role of the (P)RR. We then address the origin of renal AGT and discuss the various enzymes involved in kidney angiotensin generation and metabolism, including their local expression, as well as the three major angiotensin receptors. Next, the renal RAS in hypertension, kidney disease, and metabolic disease is discussed, critically addressing the role of urinary AGT as a biomarker reflecting renal RAS activity. Finally, the review summarizes how novel and existing drugs (RAS blockers, ACE2 activators, ARNI, COX inhibitors, and SGLT2 inhibitors) affect the renal RAS, paying particular attention to how kidney angiotensins should be measured and what their in vivo levels truly are. We do not discuss *β*-adrenergic antagonists, calcium antagonists, and diuretics since their effects on the RAS have already been extensively described (Kobori et al., 2007b).

## II. Intrarenal Angiotensin Generation: Location, Enzymes, Substrates, and Receptors

### A. Prorenin, Renin, and (Pro)renin Receptor: Juxtaglomerular Cells

**Fig. 1:**
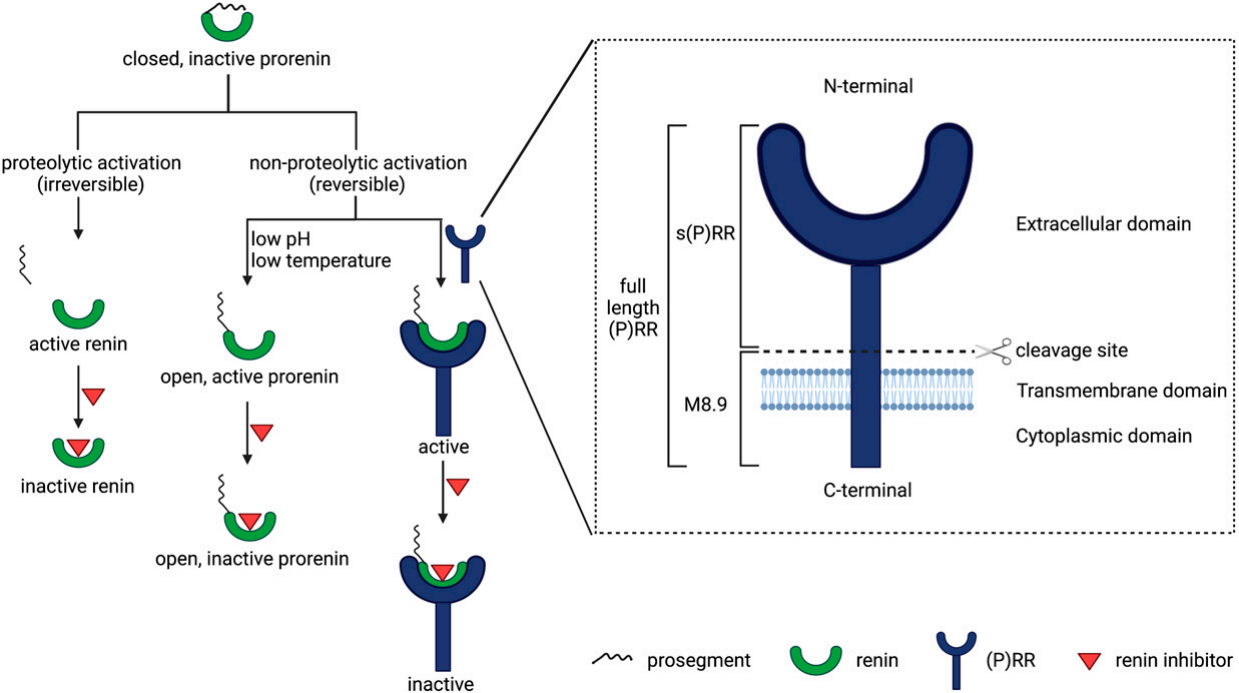
Overview of all (pro)renin forms, prorenin activation, and the (pro)renin receptor in all its roles.

Prorenin is the inactive precursor of renin. It contains a 43-amino-acid prosegment covering the active site (Danser and Deinum, 2005). Although both renin and prorenin are synthesized and secreted by JG cells, only renin is capable of cleaving angiotensin (Ang) I from AGT. Yet, the kidneys secrete much more prorenin, and generally the circulating prorenin levels are 10-fold higher than those of renin (Danser et al., 1998). The latter is also related to the fact that other organs, including the ovaries, testes, and adrenals (Krop and Danser, 2008), secrete prorenin as well. Prorenin can be activated in a proteolytic or nonproteolytic manner. Proteolytic activation occurs in renin-synthesizing cells and involves the actual removal of the prosegment by a proconvertase (e.g., kallikrein, trypsin, or cathepsin G). This process is irreversible. Acidic pH, low temperature, and receptor binding are capable of reversibly inducing a conformational change in the prorenin molecule, allowing exposure of the active site (Danser and Deinum, 2005) (Fig. 1).

Renin is synthesized primarily by JG cells located in the renal afferent arteriole. Potential additional sources of renin are renal mesangial cells, arteriolar vascular smooth muscle cells (VSMCs), interstitial pericytes, and tubuloepithelial cells (Brunskill et al., 2011; Sequeira-Lopez et al., 2015). The three major mechanisms that control renin synthesis and release into the circulation are *β*_1_-adrenergic receptors, macula densa paracrine signaling, and the renal baroreceptor. Indeed, sympathetic stimulation, decreased NaCl concentrations sensed by the macula densa, and a reduced perfusion pressure detected by renal baroreceptors upregulate renin synthesis and release (Gomez and Sequeira-Lopez, 2018). Here, the second messenger cAMP, generated by adenylate cyclase, plays a critical role, as it leads to phosphorylation of the transcription factor CREB (cAMP-responsive element-binding protein). CREB binds to the cAMP-responsive element at the renin locus regulatory region. Access to this region is determined by the histone acetyltransferases CREB-binding protein and p300, which elicit the deposition of acetylation of histones around the cAMP-responsive element, thus resulting in the displacement of nucleosomes and opening of chromatin for CREB and allowing transcription of the renin gene (Sequeira-Lopez and Gomez, 2021). Importantly, Ang II inhibits renin synthesis and release by JG cells via the AT_1_ receptor, thereby creating a negative feedback loop within the RAS.

Renin-expressing cells emerge in renal and extrarenal tissues during embryonic development (Sequeira López et al., 2004). Renin progenitor cells consisting of JG cells, mesangial cells, VSMCs, and pericytes derive from forkhead box protein D1 (Foxd1)-expressing progenitors within the stromal compartment (Sequeira-Lopez et al., 2015). In fetal mammalian kidneys, renin-producing cells exist along the afferent arterioles of the glomerulus and in the mesangium. In adult mammals, renin-producing cells are confined to the JG area, whereas those in the arterioles and mesangium differentiate into VSMCs and mesangial cells (Sequeira-Lopez and Gomez, 2021). Given the function of the RAS, renin-producing cells are vital for survival and homeostatic maintenance in the body. Further, they also play an essential role in the morphogenesis of the renal arteriolar tree and the regeneration of injured glomerular cells (Gomez and Sequeira-Lopez, 2018; Guessoum et al., 2021; Sequeira-Lopez and Gomez, 2021). Three major factors control the differentiation and maintenance of renin cells. In addition to the aforementioned cAMP/CREB-binding protein/p300 pathway, the Notch/RBP-J pathway and the transcription factor Foxd1 are also crucial for the development of renin cells and kidney vasculature (Lin et al., 2014; Castellanos-Rivera et al., 2015). Ablation of RBP-J in renin-producing cells or Foxd1^+^ stromal cells caused a reduction in the number of renin-producing cells and abnormal renal vasculature (Castellanos Rivera et al., 2011; Lin et al., 2014; Castellanos-Rivera et al., 2015). Furthermore, renin cells rely on the interaction with adjacent cells to maintain their phenotype. Connexin 40 and *β*1-integrin play an important role in the communication between renin cells and other cell types (Sequeira-Lopez and Gomez, 2021). Connexin 40 helps to anchor renin cells to JG sites and to control renin synthesis and release. Conditional deletion of Connexin 40 in renin-expressing cells led to mislocalization of JG renin cells and excessive secretion of renin, resulting to malignant hypertension (Wagner et al., 2007). *β*1-integrin is required for the development and function of renin cells, and its absence caused a marked decrease in the number of renin cells and vascular alterations (Mohamed et al., 2020).

Renin lineage cells retain the plasticity and developmental memory to reexpress renin when faced with homeostatic threats such as hypotension, fluid-electrolyte imbalance, and hypoxia (Sequeira López et al., 2004). Indeed, in response to homeostatic challenges, not only JG cells start to synthesize and secrete more renin, but simultaneously VSMCs along the renal arterioles transform into renin cells by remodeling their chromatin to carry activating epigenetic marks such as H3K27Ac, thereby allowing binding of transcription factors (Guessoum et al., 2021). Super-enhancers unique to renin cells serve as chromatin sensors for the maintenance of renin-expressing cell identity and memory (Sequeira-Lopez and Gomez, 2021). Once homeostasis is restored, the transformed cells return to their VSMC phenotype. However, when homeostasis fails to be restored (e.g., due to chronic stimuli such as RAS blockade, hypotension, and salt depletion), the progressive transformation of arteriolar cells occurs constantly and the renin program is inappropriately overactivated, leading to concentric vascular hypertrophy (Gomez and Sequeira-Lopez, 2018; Guessoum et al., 2021; Sequeira-Lopez and Gomez, 2021). Hence, the deletion of RAS genes or chronic RAS inhibition in both mice and humans causes concentric arterial and arteriolar hypertrophy accompanied by the accumulation of renin cells (Oka et al., 2017; Guessoum et al., 2021; Watanabe et al., 2021). Ablation of renin cells by either conditional *β*1-integrin deletion or diphtheria toxin prevents this phenomenon (Pentz et al., 2004; Watanabe et al., 2021), indicating that renin cells are responsible for vascular hypertrophy.

The (P)RR, also known as ATP6AP2, is a 350-amino-acid receptor protein, which was cloned by Nguyen et al. (2002). It is composed of a N-terminal extracellular domain, a transmembrane domain, and a C-terminal cytoplasmic domain (Burcklé and Bader, 2006). There are three forms of (P)RR: the 39 kDa full-length (P)RR, the 28 kDa soluble (P)RR [s(P)RR], and the 8.9 kDa M8.9 (Fig. 1). Soluble (P)RR is generated via cleavage of the full-length (P)RR by either furin (Cousin et al., 2009), a disintegrin and metalloproteinase (ADAM)-19 (Yoshikawa et al., 2011), or site 1 protease (Nakagawa et al., 2017). The (P)RR is ubiquitously expressed (Nguyen et al., 2002) and in the kidney occurs in the macula densa, mesangial cells, podocytes, proximal tubule, and CD (Advani et al., 2009; Gonzalez et al., 2011; Gonzalez et al., 2013). Its name is based on the observation that it binds both renin and prorenin and allows prorenin to undergo the conformational change described above, rendering it active. Since prorenin binds with greater affinity (Batenburg et al., 2007), the (P)RR was initially welcomed as the receptor of prorenin that finally explained why we have such high prorenin levels: the inactive prorenin would display activity at tissue sites, allowing angiotensin generation in case of local (P)RR expression. Exciting data were obtained with the putative (P)RR antagonists handle region peptide and PRO20 (Ichihara et al., 2004), which both mimic the parts of the prorenin prosegment that are believed to interact with the (P)RR. Rigorous testing of these antagonists never occurred, and until today no convincing data exist that support that these tools truly represent (P)RR antagonists. In fact, the few studies that did investigate this found no evidence for any antagonistic properties of handle region peptide (Feldt et al., 2008; te Riet et al., 2014). A further complicating factor is that the binding affinity of the (P)RR for both renin and prorenin is many orders of magnitude below its in vivo levels, thus making a meaningful interaction in vivo unlikely (Batenburg et al., 2011), except possibly at sites where prorenin is produced locally. Simultaneously, over the last 10–15 years, multiple alternative RAS-independent functions for the (P)RR as a subunit of vacuolar H^+^-adenosine triphosphatase (ATPase) have been discovered related to autophagy (Kinouchi et al., 2010), Wnt/*β*-catenin signaling, embryonic development, and cell differentiation (Cruciat et al., 2010), as well as lipid metabolism and energy homeostasis (Ren et al., 2018). Moreover, (P)RR deletion is lethal (Kinouchi et al., 2010), whereas RAS gene deletion is not. As a consequence, the concept that the (P)RR is the endogenous receptor of prorenin at extrarenal sites is now being reevaluated (Sun et al., 2017).

Nevertheless, there may still be indirect associations between the (P)RR and (pro)renin. For instance, Riquier-Brison et al. (2018) observed a link between the (P)RR and renin release, involving prostaglandin E2 (PGE2) generation by COX-2. In line with this finding, macula densa (P)RR conditional knockout (KO) mice displayed diminished plasma renin concentrations and low blood pressure, particularly in the setting of low salt and RAS blockade (Riquier-Brison et al., 2018). Wang et al. (2021) recently confirmed this view. Furthermore, (P)RR ablation is detrimental to kidney development and function (Yosypiv, 2017): nephron progenitor-specific (P)RR deficiency reduced the number of nephrons and resulted in small cystic kidneys (Song et al., 2016), podocyte-specific (P)RR KO led to severe proteinuria and kidney failure (Oshima et al., 2011), and ureteric bud-specific (P)RR deletion induced renal hypodysplasia and defects in urine concentration and acidification capacity (Song et al., 2013). These phenomena suggest that the (P)RR, by interfering with kidney function and fluid homeostasis, is likely to affect JG cell (pro)renin release. Finally, megalin-mediated (pro)renin reabsorption in the proximal tubule involves (P)RR-dependent endosomal acidification (Sun et al., 2020a), as will be discussed below. Taken together, although direct (P)RR-(pro)renin interaction at multiple sites in the body now seems unlikely, the (P)RR and the RAS appear to be indirectly linked at multiple levels.

### B. Collecting Duct (Pro)renin and (Pro)renin Receptor

**Fig. 2:**
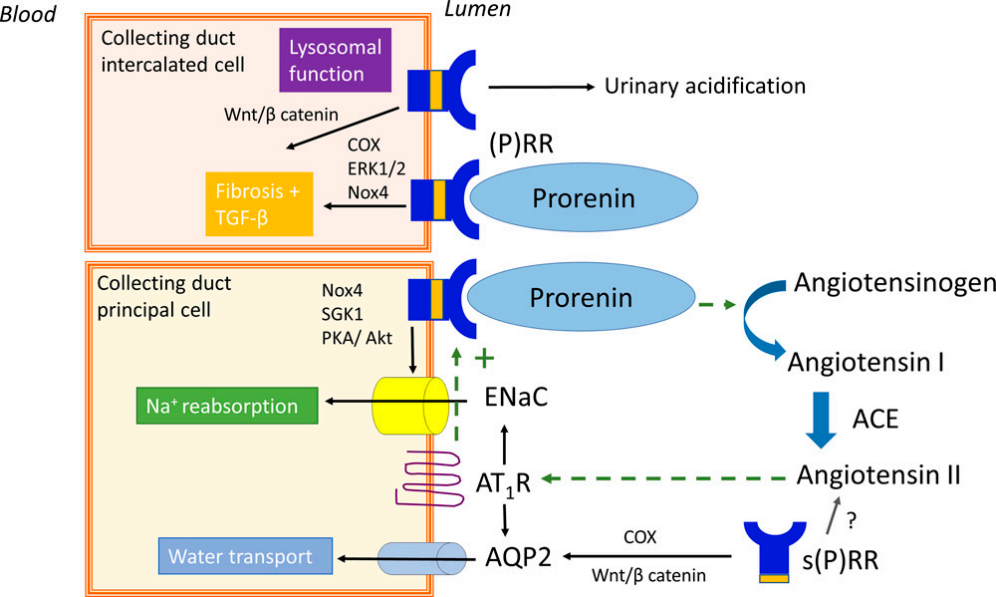
Proposed role of collecting duct prorenin and the (pro)renin receptor [(P)RR]. Dotted lines show the positive feedback loop of angiotensin II-dependent prorenin and (P)RR synthesis. Prorenin binding to the (P)RR has been linked to water transport, sodium reabsorption, and fibrosis. Independent of prorenin, the (P)RR is involved in lysosomal function and fibrosis. Akt, protein kinase B; AQP2, aquaporin 2; Nox4, NADPH oxidase 4; PKA, proteinase kinase A; SGK1, serine/threonine-protein kinase 1.

Beyond the JG apparatus, the CD has been proposed as a major second site of (pro)renin synthesis in the kidney (Rohrwasser et al., 1999), particularly under conditions of excess local Ang II such as diabetes and hypertension (Prieto-Carrasquero et al., 2004; Kang et al., 2008). According to this concept, (pro)renin is produced in the principal cells of the CD (Rohrwasser et al., 1999) and secreted in the tubular lumen. It may thus appear in urine (Liu et al., 2011b) along with filtered (pro)renin from the systemic circulation (Roksnoer et al., 2016a). Obviously, to allow prorenin to display activity at this location, either proteolytic cleavage to renin is required or it should undergo a conformational change (Fig. 1). In the absence of any known prorenin-renin converting enzyme in the CD, it has been argued that the (P)RR may help to achieve this conformational change (Nguyen and Muller, 2010). A key finding in support of this notion is that the (P)RR is predominantly localized to the CD, specifically on the luminal membrane of the intercalated cells (Advani et al., 2009). Prorenin synthesized from the principal cells might thus act in a paracrine manner on the intercalated cell (P)RR to generate Ang I, provided that AGT is available and that the local prorenin levels are sufficiently high. Additionally, (pro)renin bound to the (P)RR has been reported to activate signaling pathways such as p38 mitogen-activated protein kinase (MAPK) and extracellular signal-regulated kinase 1/2 (ERK1/2) (Nguyen et al., 2002; Batenburg et al., 2011). A further possibility is that s(P)RR exerts effects of its own (Cousin et al., 2009; Fang et al., 2017; Nakagawa et al., 2017), whereas simultaneously there may be a role for the (P)RR as an accessory protein for vacuolar H^+^-ATPase (Ludwig et al., 1998) as discussed earlier. Consequently, CD prorenin and the (P)RR may modulate CD cell function through both RAS-dependent and -independent pathways (Fig. 2).

Several factors have been shown to modulate CD (pro)renin and (P)RR synthesis (Table 1), including Ang II, sodium intake, and hyperglycemia. Under cell culture conditions, Ang II stimulates CD (pro)renin and (P)RR expression via the AT_1_ receptor (Prieto-Carrasquero et al., 2004; Gonzalez et al., 2011). The Ang II–mediated effects on CD (pro)renin synthesis are contrary to what occurs in the JG apparatus where Ang II inhibits renin synthesis. Similarly, excess systemic Ang II increases renal medullary (P)RR transcript and urinary excretion of the s(P)RR (Gonzalez et al., 2011). Notably, Ang II–stimulated (P)RR expression appears to be mediated via COX-2 through PGE2 and its EP4 receptor (Gonzalez et al., 2014a; Wang et al., 2014). Thus, a feed-forward system might exist within the CD wherein Ang II effects are amplified in the lumen via increased synthesis of CD (pro)renin and/or (P)RR. This mechanism might explain, at least in part, why low sodium intake augments CD (pro)renin and (P)RR expression (Rohrwasser et al., 1999; Quadri and Siragy, 2014). Further, independent of Ang II, sodium depletion has also been shown to modulate CD (P)RR expression via other pathways such as the glycogen synthase kinase 3*β*/nuclear factor of activated T cell 5/sirtuin-1 pathway and the cGMP/protein kinase G pathways (Quadri and Siragy, 2014). Hyperglycemia is another important regulator of (pro)renin synthesis, and the CD has been suggested to be the origin of the elevated circulating prorenin levels in diabetes (Kang et al., 2008). Simultaneously, circulating renin levels are low in this condition, whereas urinary renin is elevated (Hollenberg et al., 2011; van den Heuvel et al., 2011). Although the latter would be in accordance with CD (pro)renin release in diabetic conditions, a recent study found that the increased urinary renin levels in diabetes were actually due to increased glomerular filtration and impaired proximal tubular reabsorption via megalin (Tang et al., 2019). Moreover, prorenin is usually undetectable in urine, except under conditions where megalin-mediated reabsorption is impaired (van den Heuvel et al., 2011; Roksnoer et al., 2016a). Combined with the fact that Tang et al. (2019) were unable to demonstrate migration of renin lineage cells to the CD, the question arises as to whether local (pro)renin synthesis in vivo truly occurs in the CD (Pohl et al., 2010; Roksnoer et al., 2016a; Sun et al., 2019). An alternative explanation might be that reduced megalin binding of excess filtered prorenin allows its accumulation in the CD under diabetic conditions (Sun et al., 2019; Tang et al., 2019). This may also be true under conditions where Ang II is infused since high Ang II levels will impair glomerular filtration (increasing (pro)renin filtration), whereas Ang II simultaneously downregulates megalin (Hosojima et al., 2009), thus resulting in increased exposure of the CD to filtered (pro)renin.

**TABLE 1:** (Pro)renin and the (pro)renin receptor [(P)RR] in the collecting duct: synthesis and effects, including effects that involve the soluble s(P)RR

Synthesis	
(Pro)renin	↑ after Ang II (Prieto-Carrasquero et al., 2004)
	↑ after low sodium (Rohrwasser et al., 1999)
	↑ after hyperglycemia (Kang et al., 2008)
(P)RR	↑ after Ang II (Gonzalez et al., 2011, 2014b; Wang et al., 2014)
	↑ after low sodium (Quadri and Siragy, 2014)
	↑ or = after hyperglycemia (Ichihara et al., 2008; Matavelli et al., 2010)
Effects (P)RR	
Water transport	↑ via Ang II or Ang II–independent (Song et al., 2013; Ramkumar et al., 2015; Lu et al., 2016b; Wang et al., 2016)
Sodium reabsorption	↑ partly via Ang II (Ramkumar et al., 2015, 2016b, 2018; Lu et al., 2016a,b; Quadri and Siragy, 2016)
Fibrosis	↑ Ang II–independent (Reyes-Martinez et al., 2019)
Urinary acidification	↑ (pro)renin-independent (Trepiccione et al., 2016)
Effects s(P)RR	
Water transport	↓ in a polyuria model (Lu et al., 2016b; Wang et al., 2019)
Sodium reabsorption	↑ via ENaC (Wang et al., 2020; Feng et al., 2021)
Blood pressure	↑ during Ang II infusion (Ramkumar et al., 2021)

CD (P)RR expression changes with hyperglycemia are inconsistent, with some studies reporting increased (P)RR expression in diabetes (Matavelli et al., 2010) and others finding no difference (Ichihara et al., 2008). A crucial issue remains as to whether the augmented luminal (pro)renin levels under diabetic conditions, derived from either the systemic circulation and/or CD synthesis (Tang et al., 2019), are sufficiently high to allow local angiotensin generation.

A wide range of studies suggest that CD (pro)renin and (P)RR have the ability to modulate CD function (Table 1), including water transport (Ramkumar et al., 2015; Lu et al., 2016b), sodium homeostasis and blood pressure (Lu et al., 2016a; Ramkumar et al., 2016b, 2018), fibrosis (Reyes-Martinez et al., 2019), and acid-base balance (Ludwig et al., 1998; Lu et al., 2013; Daryadel et al., 2016; Trepiccione et al., 2016). Despite initial observations that the effect on water transport involved Ang II–mediated effects related to (P)RR-(pro)renin interaction (Song et al., 2013; Ramkumar et al., 2015; Lu et al., 2016b; Wang et al., 2016), further analyses demonstrated that this was rather due to CD (P)RR effects involving activation of COX and the Wnt/*β*-catenin pathway (i.e., this was unrelated to Ang II) (Lu et al., 2016b; Wang et al., 2016). Concurrently, CD prorenin acting via the (P)RR can modulate sodium balance via changes in epithelial Na^+^ channel (ENaC) abundance and activity (Ramkumar et al., 2015; Lu et al., 2016b; Ramkumar et al., 2016b, 2018). These effects have been attributed to NADPH oxidase 4–derived H_2_O_2_ (Lu et al., 2016a), serum- and glucocorticoid-regulated kinase-1 (Quadri and Siragy, 2016), and the protein kinase A and protein kinase B pathways (Ramkumar et al., 2016b) and might partly involve Ang II. In accordance with early in vitro studies in VSMCs (Batenburg et al., 2011), prorenin binding to the (P)RR in the kidney has also been suggested to upregulate the release of profibrotic factors like transforming growth factor *β*1 (TGF*β*1) through activation of MAPK/ERK1/2, COX, and NADPH oxidase 4 (Reyes-Martinez et al., 2019). Furthermore, the role of the (P)RR as an accessory protein for vacuolar H^+^-ATPase (Advani et al., 2009; Lu et al., 2013; Daryadel et al., 2016) needs to be considered since this ATPase is important for urinary acidification, lysosomal function, and autophagy (Trepiccione et al., 2016). Hence, it remains to be determined whether the functional significance of the CD (P)RR goes beyond modulation of vacuolar H^+^-ATPase (Meima and Danser, 2011).

Finally, recent evidence suggests that the s(P)RR can modulate CD function (Table 1). Recombinant s(P)RR activates Wnt/*β*-catenin signaling in CD cells and alleviates polyuria in several rodent models with polyuria (Lu et al., 2016b; Wang et al., 2019). Similarly, s(P)RR can regulate CD ENaC function (Wang et al., 2020; Feng et al., 2021) and blood pressure (Feng et al., 2021; Ramkumar et al., 2021). Further, elevated plasma s(P)RR levels have been described in humans and mice with hypertension (Morimoto et al., 2014) and other sodium-retentive states such as heart failure and kidney disease (Fukushima et al., 2013). Conversely, the loss of s(P)RR in mice reduced blood pressure at baseline and decreased Ang II–induced hypertension and kidney injury (Ramkumar et al., 2021). Since the s(P)RR retains the prorenin-binding site, it has been suggested that s(P)RR effects on CD function might involve Ang II signaling (Feng et al., 2021). However, as shown recently, loss of the s(P)RR did not alter the systemic or renal Ang II levels (Ramkumar et al., 2021), in agreement with the observation that the prorenin levels required for such interaction do not occur in vivo. This indicates that s(P)RR acts via RAS-independent mechanisms such as by modulating endothelial cell function (Fu et al., 2021; Ramkumar et al., 2021), and/or pathways within the CD.

### C. Liver-Derived AGT Is the Source of Kidney Angiotensins: Importance of Megalin

Because the proximal straight tubule (S3 segment) highly expresses AGT mRNA, local transcriptional activity of the AGT gene was thought to be a major determinant of kidney Ang II generation. Interestingly, Ang II increased renal AGT mRNA via its AT_1_ receptor, thus potentially creating a feed-forward loop that might contribute to the development of diabetic kidney disease (DKD), also because high glucose similarly increases renal AGT mRNA (Gonzalez-Villalobos et al., 2008; Nishiyama et al., 2008; Nishiyama and Kobori, 2018). However, studies using tissue-specific *Agt* KO mice demonstrated that the liver is the major source not only of plasma AGT but also renal AGT. Indeed, liver-specific *Agt* KO decreased plasma AGT by more than 95%, and similar decreases were seen for AGT and Ang II in the kidney (Matsusaka et al., 2012). Recent studies making use of hepatocyte-directed small interfering RNA (siRNA) for AGT in rats displaying hypertension and renal injury [spontaneously hypertensive rats (SHRs), deoxycorticosterone acetate-salt-treated hypertensive rats, and five-sixths nephrectomy rats], or liver-targeted AGT antisense oligonucleotides in normotensive cynomolgus monkeys yielded the same conclusions (Uijl et al., 2019, 2021; Bovée et al., 2021; Kukida et al., 2021).

In contrast, proximal tubule–specific *Agt* KO did not affect renal AGT and Ang II content, nor kidney histology, blood pressure, and urinary sodium excretion (Matsusaka et al., 2012). Urinary AGT protein was decreased by approximately 50% in proximal tubule–specific *Agt* KO mice, indicating that AGT protein produced in the S3 segment is directly excreted into urine. It should be noted that lack of an abnormal basal gross phenotype does not mean lack of functional significance because there may be compensation by other factors. In addition, effects of Ang II infusion or diabetes have not been tested in these mice. Ramkumar et al. (2016a) reported that nephron-specific *Agt* KO mice showed lower blood pressure, but since in these mice the *Agt* gene was also disrupted in the liver, this phenotype likely represents combined hepatic and renal AGT suppression.

Immunostaining detected AGT protein in proximal convoluted tubules (S1 and S2 segments), whereas AGT mRNA is detected in proximal straight tubules (S3 segment). AGT is stained in a granular pattern, similarly to that of albumin. This indicates that AGT in the S1 and S2 segments, like albumin, is reabsorbed from tubular fluid. Indeed, a great number of proteins, including albumin, is reabsorbed in the proximal tubules via the multiligand receptor megalin. *Megalin* KO mice revealed that AGT staining was dependent on megalin (Pohl et al., 2010). For unknown reasons, the glomerular permeability of AGT is approximately one-fourth that of albumin (Nakano et al., 2012) despite the fact that the molecular mass of AGT (53 kDa) is lower than that of albumin (66 kDa) and lacks negative charge. Nevertheless, the above findings indicate that a small amount of plasma AGT is filtered through the glomerulus and reabsorbed by the proximal convoluted tubule (S1 and S2 segments) via megalin. Data from humans displaying megalin dysfunction fully concur with this concept (Roksnoer et al., 2016a).

This notion leads to the possibility that disruption of the glomerular filtration barrier increases delivery of AGT to the tubules and might thus enhance kidney Ang II generation. In fact, when the glomerular barrier was disrupted by inducing podocyte injury, the amount of reabsorbed AGT was markedly increased, concurrently with an increase in urinary AGT (Matsusaka et al., 2012). Importantly, the renal Ang II content was also increased, independently of renal renin. In accordance with this finding, Matsuyama et al. (2021) showed that the renal Ang II levels correlated with the glomerular filtration of AGT in doxorubicin (Adriamycin) nephropathy rats. Studies using tissue-specific *Agt* KO mice again showed that the increased renal Ang II generation by podocyte injury was completely dependent on liver-derived AGT (Matsusaka et al., 2014). In addition, ureteral obstruction attenuated the increase of AGT protein and Ang II in kidneys with podocyte injury (Okabe et al., 2015), indicating that the major source of increased renal Ang II in podocyte injury is filtered AGT. Although podocyte injury also increased AGT mRNA in proximal tubular cells, this did not contribute to renal Ang II because proximal tubule–specific *Agt* KO did not decrease renal Ang II.

Earlier studies showed that infusion of Ang II into rodents increased renal AGT mRNA, renal and urinary AGT protein, and renal Ang II content (Navar et al., 2002; Kobori et al., 2007b; Gonzalez-Villalobos et al., 2008). The increase in renal AGT mRNA was thought to be the cause of the increase in renal Ang II. However, as discussed above, the contribution of renal AGT to renal Ang II synthesis, both under control and nephrotic conditions, is virtually absent. Tissue-specific *Agt* KO mice are needed to verify whether renal AGT contributes to renal Ang II synthesis after Ang II infusion. A possible alternative mechanism is that Ang II increases glomerular capillary pressure, which may increase glomerular filtration of plasma AGT and tubular reabsorption of AGT. Supporting this hypothesis, it was shown that Ang II infusion increased granular staining of AGT in S1 and S2 segments (Gonzalez-Villalobos et al., 2008).

**Fig. 3:**
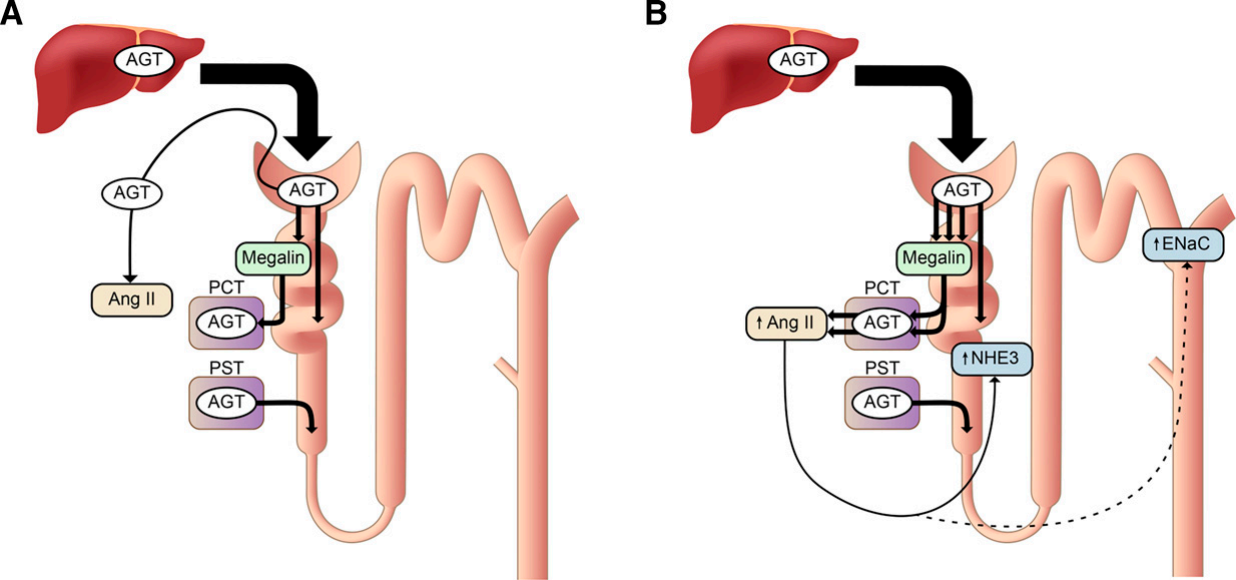
Proposed mechanism of renal angiotensin (Ang) II generation and its function. (A) Under normal conditions, a portion of plasma AGT is filtered through the glomeruli and reabsorbed by proximal convoluted tubules (PCTs, S1, and S2 segments) via megalin. Its contribution to renal Ang II generation is marginal. AGT is synthesized in proximal straight tubules (PSTs, S3 segment), but this AGT also makes little contribution to renal Ang II formation, although it may appear in urine. Renal Ang II is probably generated from liver-derived AGT in the capillary lumen or interstitium. (B) In nephrotic syndrome, podocyte injury increases glomerular leakage of plasma AGT. The filtered AGT is reabsorbed by proximal tubules via megalin and thereafter converted to Ang II. The increased renal Ang II content may contribute to sodium retention, possibly by activating Na^+^/H^+^ exchanger 3 (NHE3) and the epithelial Na^+^ channel (ENaC) (Koizumi et al., 2019).

The above results suggest that AGT protein reabsorbed via megalin contributes to Ang II generation at renal tissue sites. This hypothesis was tested using proximal tubule–specific *megalin* KO mice (Koizumi et al., 2019). As expected, renal AGT protein was markedly decreased and urinary AGT excretion was increased in these mice. However, their intrarenal Ang II content was comparable to that in control mice, without changes in blood pressure and urinary sodium excretion. This implies that the AGT reabsorbed by the proximal tubular cells via megalin in healthy, normotensive mice does not contribute to local Ang II generation (Fig. 3). Therefore, under normal circumstances, the major source of renal Ang II is probably AGT within the capillary lumen or interstitium of the kidney. Indeed, diffusion of AGT from blood to the interstitial space is known to occur (de Lannoy et al., 1997). In contrast, Ye et al. (2019) reported that suppression of megalin by antisense oligonucleotides decreased renal Ang II by 70% in mice without podocyte injury. A unifying concept might be that in Koizumi’s study, renal renin was sufficiently upregulated to restore renal Ang II, whereas in Ye’s study it was not (Sun et al., 2020b). If true, this would imply that reabsorbed AGT does contribute after all to renal Ang II generation under normal conditions. However, neither study reported renal renin levels, and thus this question remains to be answered.

When podocytes are severely injured, a large amount of plasma AGT leaks into the tubular lumen. This AGT will be avidly reabsorbed by proximal tubular cells via megalin. In this situation, *megalin* KO further increased urinary AGT as expected (Koizumi et al., 2019). The kidneys of these animals showed massive proteinaceous casts. Immunostaining revealed that most AGT staining occurred on these proteinaceous casts, whereas proximal tubular cells were devoid of staining of AGT. The renal Ang II content in the *megalin* KO mice with podocyte injury was significantly reduced. These results indicate that, in the nephrotic state, AGT filtered through the glomerulus and reabsorbed via megalin by the proximal tubule is converted to Ang II (Fig. 3), whereas AGT in the tubular lumen is not.

The suppression in renal Ang II was paralleled by a reduction in renal sodium reabsorption in *megalin* KO mice with podocyte injury. Podocyte injury does not increase blood pressure but markedly increases urinary sodium reabsorption, which causes retention of extracellular fluid and edema formation, two hallmarks of the nephrotic syndrome. In *megalin* KO mice with podocyte injury, this increase in urinary sodium reabsorption was less prominent. In addition, the cleaved forms of *α*- and *γ*-subunits of ENaC, which reflect the activation of this channel, and membrane-bound Na^+^/H^+^ exchanger 3 (NHE3) were increased in *megalin*-intact nephrotic mice, and this increase was significantly attenuated in nephrotic *megalin* KO mice (Koizumi et al., 2019). Collectively, these data suggest that increased renal Ang II by podocyte injury may contribute to sodium retention by activating ENaC and NHE3. The renal Ang II generation from the leaked plasma AGT may be a mechanism underlying the overfill hypothesis of nephrotic syndrome (i.e., proteinuria primarily stimulates sodium reabsorption).

It should be noted, however, that these data were obtained in an extreme condition, where a very severe nephrotic syndrome was rapidly developing. Translating the role of megalin in renal Ang II generation to all proteinuric conditions is therefore not yet possible. In this regard, it was reported that *megalin* KO mice with mild proteinuria show more activated *α*-subunit of ENaC, without changes in renin and aldosterone (Kastner et al., 2009). This opposes the findings of the observations in *megalin* KO mice with severe nephrotic syndrome. It might involve ENaC activation by proteases in urine, although this was described for the *γ*-subunit of ENaC only (Hinrichs et al., 2020). If confirmed, however, sodium reabsorption in nephrotic syndrome and other proteinuric conditions might be treated with an ENaC blocker (amiloride) rather than RAS blockers.

The next obvious question is where and how the reabsorbed AGT is converted to Ang II. Based on in vitro data in cultured opossum kidney cells, Pohl et al. (2010) reported that a portion of AGT is transcytosed to the basolateral membrane and released into the interstitium. Renin has a smaller molecular mass (43 kDa) and a larger glomerular sieving coefficient than AGT, whereas its glomerular leakage is similarly increased after podocyte injury (Roksnoer et al., 2016a). As discussed, renin is also reabsorbed via megalin, and this is true for prorenin as well (Sun et al., 2020a). Taken together, these data suggest that Ang II is likely formed in proximal tubules, although the exact location of this formation (tubular lumen or peritubular interstitium) still needs to be identified. Future studies should also investigate how hepatic AGT ensures local generation of Ang II at the level of the CD, which is an important site for controlling sodium reabsorption in nephrotic syndrome. Given the well known beneficial effects of RAS blockers on tubulointerstitial injury, it now seems likely that this relies on interference with Ang II generated via a megalin-dependent mechanism. This may offer new insights for the treatment of this condition.

### D. ACE, Chymase, and the Site of Angiotensin Generation

Renal ACE is abundantly expressed in the brush border of the proximal tubule (apical and basolateral) but can also be found in vascular endothelial cells, mesangial cells, and distal nephron segments (Metzger et al., 1999; Gonzalez-Villalobos et al., 2013). ACE has been reviewed before in this journal (Bernstein et al., 2012). It is a peptidyl dipeptidase with two catalytic domains (N and C), of which the C-domain is responsible for Ang I-II conversion (van Esch et al., 2005; Arendse et al., 2019). Classically, ACE is the enzyme responsible for Ang II formation from Ang I. The renoprotective effects of ACE inhibitors (ACEIs) demonstrated in multiple clinical trials are in full agreement with this concept. Both the human and rat glomerular vascular beds are remarkably devoid of endothelial ACE compared with other renal and extrarenal vascular beds (Metzger et al., 2011). This is believed to afford protection against excess Ang II–mediated renal vasoconstriction, keeping renal blood flow high.

Recently, chymase, discovered more than 30 years ago in human heart homogenates (Urata et al., 1990), has been put forward as a major Ang I-II converting enzyme in the kidney (Park et al., 2010; Kaltenecker et al., 2020). A major reason for invoking additional converting enzymes is the so-called Ang II escape (i.e., the return of renal Ang II levels to baseline levels during prolonged ACE inhibition). If true, one would expect the clinical effectiveness of AT_1_ receptor blockers (ARBs) to be better than that of ACEIs, although in reality they are similar (Wu et al., 2013; Thomopoulos et al., 2017). Moreover, renal Ang II levels are negligible in ACE KO animals (Alexiou et al., 2005), and chymase inhibition was ineffective in patients with DKD (Rossing et al., 2021). Here it is important to stress that chymase is largely located intracellularly and thus normally may not ‘see’ Ang I, except when tissue is homogenized (Kaltenecker et al., 2020). A much more logical reason for the return of renal Ang II levels is therefore that ACE inhibition will result in a rapid renin rise due to the drop in Ang II. Additional counterregulation may occur via the upregulation of ACE. However, such renal ACE upregulation is usually of modest proportion (2- to 3-fold) (Gonzalez-Villalobos et al., 2013) compared with the many 100-fold upregulation in renin levels that the body is capable of by recruiting additional renin-synthesizing cells (Balcarek et al., 2014). For instance, in case of 90% ACE inhibition, a 10-fold rise in renin is sufficient to allow Ang II levels to return to normal, whereas a 2-fold rise in ACE would have negligible consequences.

Early studies making use of infusions of ^125^I-labeled angiotensins (i.e., allowing the detection of ^125^I-angiotensins at levels that do not affect blood pressure) revealed that there is substantial uptake of circulating ^125^I-Ang II at renal tissue sites: its steady state tissue levels corresponded to ≈4 times the steady-state plasma levels of ^125^I-Ang II (van Kats et al., 2001a; Zhuo et al., 2002). Uptake depended entirely on binding to AT_1_ receptor (van Kats et al., 1997; Zhuo et al., 2002). At the same time, the levels of endogenous Ang II at renal tissue sites were up to 100 times higher than the plasma levels of endogenous Ang II, and thus it could be calculated that, despite significant uptake of circulating Ang II, the majority (>95%) of renal Ang II had been synthesized locally by renal ACE (Schalekamp and Danser, 2006a,b). Infusion studies with ^125^I-Ang I confirmed that this was not due to the conversion of plasma-derived Ang I. In other words, these studies unequivocally proved that both Ang I and Ang II are produced locally at renal tissue sites and that renal Ang II is derived from locally generated Ang I and not from plasma-derived Ang I. Furthermore, although most, if not all, renal Ang II was cell associated (i.e., either bound to membrane AT_1_ receptor or located intracellularly) (van Kats et al., 2001b), this was not due to intracellular angiotensin generation but the consequence of AT_1_ receptor binding followed by Ang II–AT_1_ receptor internalization. Indeed, there was no cell-associated Ang II in AT receptor KO mice, despite the tremendous rises in renin that occur in such animals (van Esch et al., 2010b). Therefore, renal angiotensin production occurs extracellularly, in the renal interstitial space, and/or on the surface of renal cells.

### E. ACE2, Neprilysin, and Other Angiotensinases

**Fig. 4:**
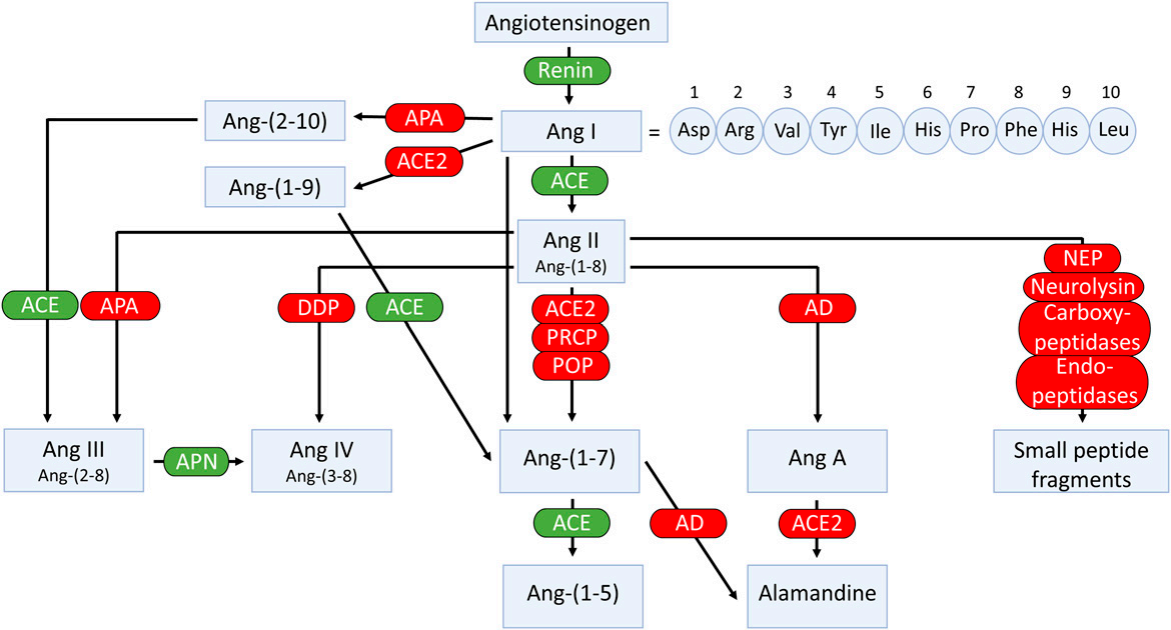
Enzymatic pathways involved in the generation and metabolism of angiotensins. AD, aspartate decarboxylase.

In addition to the classic cascade resulting in the formation of the octapeptide Ang II [=Ang-(1-8)] from the decapeptide Ang I [=Ang-(1-10)] by ACE, ACE2 can cleave Ang I to Ang-(1-9), which is then converted to Ang-(1-7) by ACE (Fig. 4). Alternatively, Ang-(1-7) can be formed directly from Ang I by neprilysin (Domenig et al., 2016). Ang II degradation involves aminopeptidase A (APA), neprilysin, dipeptidyl aminopeptidase III (DDP), aspartate decarboxylase, and neurolysin (McDonald et al., 1974; Brown et al., 2001; Rice et al., 2004; Marahrens et al., 2021). In the kidney, neprilysin and APA are the two most relevant Ang II–degrading enzymes. DDP generates Ang IV [=Ang-(3-8)] by cleaving the N-terminal dipeptide from Ang II and can then further cleave Ang IV (Pang et al., 2016). DDP has a much higher substrate affinity for Ang IV than for Ang II and also cleaves Ang-(1-7), but its importance for Ang II degradation within the kidney is limited. Alternatively, Ang IV can also be formed from Ang III [=Ang-(2-8)] by aminopeptidase N (Wysocki et al., 2015). Aspartate decarboxylase decarboxylates the N-terminal amino acid of Ang II (aspartic acid) to form Ang A (Jankowski et al., 2007). Ang A therefore only differs from Ang II in its first amino acid (alanine instead of aspartic acid). ACE2 can hydrolyze Ang A to form alamandine (Lautner et al., 2013). Neurolysin cleaves Ang II to Ang-(1-4) and Ang-(5-8) (Brown et al., 2001).

APA degrades Ang II to Ang III by cleaving its N-terminal amino acid. Additionally, APA converts Ang I to Ang-(2-10), which can then be converted to Ang III by ACE (Wysocki et al., 2015). Mice with a genetic deficiency of APA have increased blood pressure, possibly as a result of increased levels of Ang II (Mitsui et al., 2003; Marahrens et al., 2021). Within the kidney, strong expression of APA was found in the glomerulus and to a lesser extent in tubular structures. Immunohistochemistry observed APA costaining with the podocyte marker podocin. In isolated mouse glomeruli, the APA inhibitor amastatin reduced the degradation of Ang II, whereas the ACE2 inhibitor MLN-4760 did not (Marahrens et al., 2021). This suggests that at the glomerular level, Ang II degradation is due to APA rather than ACE2. Indeed, glomerular Ang II levels in APA KO mice were three times higher than in wild-type mice. Remarkably, APA KO reduced renal ACE, most likely to prevent excessive Ang II accumulation. Of interest, knoblike structures in the glomerular basement membrane of APA deficient mice were observed, and when challenged with Ang II, they rapidly developed albuminuria, suggesting an important role for APA in glomerular structure and function, likely exerted via the regulation of the metabolism of Ang II within the glomerulus.

Neprilysin, also known as neutral endopeptidase, degrades both Ang I and II (Rice et al., 2004). Immunohistochemistry of human kidney biopsies revealed strong neprilysin expression in glomerular epithelial cells (Domenig et al., 2016). Studies using the neprilysin inhibitor thiorphan in mouse and human kidney homogenates led to a marked reduction of Ang-(1-7) formation from Ang I (Domenig et al., 2016). Although this suggests that neprilysin is crucial for Ang-(1-7) formation at renal tissue sites, neprilysin inhibition in rats did not alter renal Ang-(1-7) levels (Roksnoer et al., 2015). In 2-kidney, 1-clip Goldblatt hypertensive mice, neprilysin was markedly reduced in the clipped kidney (Alawi et al., 2021), whereas in the kidney of diabetic db/db mice (a type 2 diabetes model), neprilysin was also significantly decreased (Alawi et al., 2020).

There are three enzymes that cleave the C-terminal amino acid (phenylalanine) of Ang II to generate Ang-(1-7): prolyloligopeptidase (POP), also known as prolylendopeptidase, prolylcarboxypeptidase (PRCP), and ACE2 (Donoghue et al., 2000; Tipnis et al., 2000; Maier et al., 2017; Serfozo et al., 2020). The latter has received the most attention, and it is indeed the most potent of the three.

POP is a serine protease that preferably hydrolyzes proline-containing peptides at the carboxyl end of proline residues (Serfozo et al., 2020). POP is preferentially an intracellular enzyme, and its contribution to renal Ang II–Ang-(1-7) conversion is modest compared with ACE2 (Serfozo et al., 2020). Indeed, in kidneys of POP KO mice, Ang-(1-7) formation from Ang II was unaltered (Grobe et al., 2013; Serfozo et al., 2020), although one study in mouse homogenates reported that POP was the main enzyme forming Ang-(1-7) from Ang II (Domenig et al., 2016). The reason for this discrepancy is unclear, but all studies agree that in the human kidney ACE2 is the dominant Ang-(1-7)–forming enzyme (Domenig et al., 2016; Grobe et al., 2013; Serfozo et al., 2020). POP KO mice have no detectable plasma Ang-(1-7) levels, suggesting that Ang-(1-7) in the circulation relies on POP (Serfozo et al., 2020).

PRCP is a serine carboxyprotease cleaving the C-terminal amino acid of various peptides where the penultimate amino acid is proline (Odya et al., 1978). It cleaves both Ang II and III. PRCP, first reported as a soluble lysosomal enzyme, can also be found extracellularly, either membrane bound or in soluble form (Jeong and Diano, 2013). In mice, PRCP deficiency increases blood pressure and results in left ventricular hypertrophy (Maier et al., 2017). Simultaneously, moderate glomerular hypertrophy and mesangial expansion occurred, without alterations in glomerular filtration rate (GFR). In 2-kidney, 1-clip Goldblatt hypertensive mice, PRCP was markedly reduced (Grobe et al., 2015).

ACE2 shares 42% homology with the metalloprotease catalytic domains of ACE (Donoghue et al., 2000; Tipnis et al., 2000). Yet, although the peptidyl dipeptidase ACE removes C-terminal dipeptides from its substrates, ACE2 functions as a monocarboxypeptidase. Ang-(1-7) can be further cleaved by ACE to form Ang-(1-5) (Rice et al., 2004). ACE2 is a zinc metalloprotease that in its full-length form has 805 amino acids and is anchored to the cell membrane by a short transmembrane domain (Turner and Hooper, 2004). The full-length membrane-bound ACE2 is expressed at high levels in multiple tissues, including kidney, testes, intestine, and heart (Donoghue et al., 2000; Tipnis et al., 2000). At very low levels it is also expressed in lung, where it is restricted to apical epithelial and ciliated cells in the upper respiratory tract and to type 2 pneumocytes in the lower respiratory tract (Serfozo et al., 2020; Ziegler et al., 2020). The importance of the location of ACE2 protein in these alveolar cells stems from it being the main cell entry receptor for severe acute respiratory syndrome coronavirus 2 (SARS-CoV-2) (Wan et al., 2020).

The soluble form of ACE2, which lacks the transmembrane domain, has 740 amino acids (Vickers et al., 2002). This soluble form is enzymatically active and is shed into blood, urine, and cerebrospinal fluid by ADAM-17 (Lambert et al., 2005). ACE2 can be also cleaved by mechanisms distinct from ADAM-17 that do not lead to the formation of the enzymatically active soluble ACE2. Specifically, transmembrane serine protease 2 (TMPRSS2), human airway trypsin-like protease, and hepsin have been reported to cleave ACE2 and to form short C-terminal fragments of about 15 kDa (Shulla et al., 2011; Heurich et al., 2014).

Within the normal kidney, full-length ACE2 is expressed abundantly in the apical membrane of the proximal tubule (Ye et al., 2004, 2006). In mice, ACE2 is also present in glomerular parietal cells and to a lesser extent in visceral epithelial cells (podocytes) (Ye et al., 2004, 2006). These findings are in agreement with human data derived from immunohistochemistry, immunofluorescence, and single cell analysis showing clear detection of ACE2 in proximal tubules and to a lesser extent in parietal epithelial cells of Bowman’s capsule (Lely et al., 2004). In human kidney homogenates, ACE2 is the main contributor to the formation of Ang-(1-7) from Ang II (Domenig et al., 2016; Serfozo et al., 2020). Alterations in kidney ACE2 can therefore affect the balance of these two peptides and may thus contribute to diabetic and hypertensive kidney disease. In diabetes, the expression of ACE2 seems to differ at the tubular and glomerular level (Lores et al., 2020). In db/db mice, glomerular ACE2 expression is decreased, whereas tubular ACE2 expression is increased (Ye et al., 2004, 2006). Similarly, in young streptozotocin (STZ)-treated diabetic rats (a type 1 diabetes model), glomerular ACE2 activity was also decreased (Leehey et al., 2008). Studies in humans with DKD confirm this pattern (Mizuiri et al., 2008; Reich et al., 2008). Kidney biopsies from patients with type 2 diabetes examined by immunohistochemistry or reverse-transcription polymerase chain reaction (RT-PCR) also showed decreased glomerular ACE2 staining/mRNA levels compared with healthy controls or controls that had focal segmental glomerulosclerosis or chronic allograft nephropathy. In these studies, however, tubular ACE2 was also found to be decreased. Both studies found glomerular ACE expression to be increased in diabetic patients.

In experimental rat models of hypertension, renal ACE2 expression is decreased (Crackower et al., 2002). These findings were confirmed in adult SHRs (Zhong et al., 2004). Increased ACE2 levels, by contrast, were found in tubules from young SHRs prior to the onset of hypertension, which then declined with the onset of hypertension (Tikellis et al., 2006). In the 2-kidney, 1-clip mouse model of hypertension, ACE2 and neprilysin expression decreased in parallel (Alawi et al., 2021). Similar to the data in animal models, biopsies from patients with hypertensive nephropathy examined by immunohistochemistry show a marked decrease of ACE2 staining in tubules (Koka et al., 2008). An immunohistochemistry study in patients with hypertensive nephrosclerosis also found tubulointerstitial ACE2 levels to be decreased, whereas glomerular ACE2 expression was not altered as compared with controls (Wang et al., 2011). In mice that underwent renal ischemia reperfusion injury, immunohistochemistry revealed reduced ACE2 staining in the corticomedullary area (Fang et al., 2013). In an animal model of ischemia reperfusion injury-induced acute kidney injury (AKI), moreover, decreased ACE2 levels were found 24 and 48 hours postsurgery (Shirazi et al., 2019).

The effect of ACE2 deficiency on kidney injury has been studied in a model of genetic ACE2 ablation. Ang II infusion in this model resulted in higher levels of blood pressure than in control mice (Gurley et al., 2006). In a model of diabetic kidney injury, ACE2 KO mice displayed increased urinary albumin excretion and more severe histopathology (Wong et al., 2007). Wysocki et al. (2014) found increased markers of oxidative stress in the kidneys of ACE2 KO mice at baseline conditions. Also, in the ischemia reperfusion model of kidney injury, ACE2 KO mice showed increased markers of oxidative stress and proinflammatory cytokines, combined with increased inflammatory cell infiltration (Fang et al., 2013). Yang et al. (2012b) studied the hindlimb-ischemia reperfusion model and found that ACE2 KO mice had more severe kidney injury than controls.

In summary, ACE2 deficiency worsens the consequences of kidney injury, whereas several kidney disease models also display lowered kidney ACE2 levels. Based on this, intact ACE2 expression is often seen as a protective factor. However, given the many substrates of ACE2, whether this relates exclusively to a disturbed Ang-(1-7)/Ang II balance remains to be proven.

### F. Angiotensin Levels in the Kidney

Measuring angiotensins is notoriously difficult, and consequently angiotensin levels reported in the literature vary widely, often by many orders of magnitude. Obtaining blood samples for angiotensin quantification requires the immediate addition of a protease inhibitor cocktail, assuring efficient stabilization of angiotensin metabolites by instantly blocking all proteases involved in their metabolism. The sampling time has to be kept as short as possible, and an immediate and efficient mixture of the blood sample and the inhibitor cocktail is essential to avoid artifacts, which are particularly challenging when collecting blood samples from mice. The importance of the sampling time is often underestimated and is likely the cause of unexpected peptide shifts despite the use of inhibitor cocktails. Tissue samples cannot be easily mixed with an inhibitor cocktail and should thus be snap-frozen in liquid nitrogen and stored at −80°C until analysis. The actual quantification often relies on commercial radioimmunoassays or ELISAs that have not been validated thoroughly and hence yield results that are entirely unreliable, particularly when using tissue homogenates with a lot of background noise (Chappell et al., 2021). Instead, prior high-performance liquid chromatography separation or liquid chromatography-tandem mass-spectrometry should be applied. Table 2 summarizes data in untreated rats where these methods were used and where a range of metabolites in appropriately collected samples was determined (Campbell et al., 1991, 1995c; Roksnoer et al., 2015; Uijl et al., 2019, 2021).

**TABLE 2:** Levels of angiotensin metabolites in kidney and blood in various rat strains

	SD	(mRen2)27	SD	SHR	(mRen2)27
	*n* = 6 mean ± S.E.M.	*n* = 8 mean ± S.E.M.	*n* = 6–8 mean (IQL range)	*n* = 8 mean ± S.D.	*n* = 5–8 mean ± S.E.M.
Kidney (fmol/g)					
Ang-(1-10)	234 ± 33	20 ± 2	564 (313–907)	413 ± 139	163 ± 69
Ang-(1-8)	338 ± 33	474 ± 40	729 (431–1892)	431 ± 44	388 ± 36
Ang-(1-7)	33 ± 8	ND	173 (65–279)	66 ± 45	20 ± 3
Ang-(1-5)	ND	ND	17 (<13–40)	16 ± 6	13 ± 4
Ang-(2-8)	<15	ND	75 (44–121)	<8	26 ± 3
Ang-(3-8)	ND	ND	<10	ND	5 ± 0
Ang-(2-10)	37 ± 5	ND	ND	ND	30 ± 15
Ang-(1-9)	113 ± 19	ND	ND	ND	42 ± 12
Blood (fmol/ml)					
Ang-(1-10)	100 ± 35	78 ± 5	141 (103–200)	375 ± 196	ND
Ang-(1-8)	47 ± 15	125 ± 9	105 (75–141)	36 ± 36	ND
Ang-(1-7)	5 ± 2	13 ± 1	<8	<7	ND
Ang-(1-5)	ND	ND	5 (3–6)	2 ± 1	ND
Ang-(2-8)	29 ± 8	ND	4 (<2.5–6)	<3	ND
Ang-(3-8)	ND	ND	4 (3–6)	4 ± 6	ND
Ang-(2-10)	34 ± 12	ND	ND	ND	ND
Ang-(1-9)	<1	ND	ND	ND	ND

IQL, interquartile; m(Ren2)27, transgenic rat overexpressing mouse *Ren2* gene; ND, not done; SD, Sprague Dawley.

Data are from Campbell et al. (1991, 1995b); Roksnoer et al. (2015); and Uijl et al. (2019, 2021) and have been determined either by radioimmunoassay after high-performance liquid chromatography separation (first two columns) or by using liquid chromatography-tandem mass-spectrometry (last three columns).

These data show that in general, Ang I and II are the main metabolites in blood, with Ang I levels usually being several-fold higher than those of Ang II. The blood levels of Ang-(1-7) and Ang III are 5- to 10-fold below those of Ang II and often below detection limit. In the kidney, Ang II is the main metabolite, with levels in the order of several 100 fmol/g (i.e., easily 10- to 20-fold higher than its levels in blood). Tissue Ang-(1-7) and Ang III levels are 5- to 10-fold lower than the tissue levels of Ang II, comparable to what is observed in blood. Assuming that Ang I has no role (i.e., that it does not bind to any of the known AT receptors), these data imply that Ang II is by far the most important active angiotensin metabolite in both blood and tissue, with only minor roles for Ang-(1-7) and Ang III. Importantly, and perhaps not surprising, angiotensin levels in other species (mice, pigs, humans), if measured appropriately, are remarkably similar to those in rats (Lawrence et al., 1990; van Kats et al., 2001a; Alexiou et al., 2005; Fisher et al., 2008; Balcarek et al., 2014). This should be the case since the affinity of Ang II for its receptors is identical in all species, and thus it would be hard to explain why tissue Ang II levels in one species would be, for instance, 10- or 100-fold higher than in another species. Moreover, no major differences in Ang I and II levels were observed between renal medulla and cortex (van Kats et al., 2001a).

Recent studies have introduced the concept of ‘equilibrium levels’ of angiotensins in serum or plasma (Pavo et al., 2021). These are obtained by incubating a sample at 37°C in the absence of inhibitors. This opposes the conditions of a plasma renin activity measurement, where plasma is incubated for a fixed amount of time in the presence of a cocktail of angiotensinase inhibitors that prevent the breakdown of Ang I (Campbell et al., 2009). Thus, only Ang I is generated under the latter assay conditions, and the amount of Ang I generated per hour is independent of the incubation period. Data are often expressed as ng Ang I per ml per hour, with Ang I generation being linear over 24 hours. Blood contains renin, AGT, ACE, and a great variety of angiotensinases. Thus, when incubating plasma or serum at 37°C, all enzymes will act simultaneously, yielding virtually every angiotensin metabolite that has been described. Here it is important to realize that this situation also exists in vivo, with one exception: ACE and all angiotensinases occur at much higher levels on endothelial cells (and potentially other vascular cells), resulting in an entirely different metabolism pattern. Indeed, the levels of ACE and ACE2 in blood represent at most a few percent of the amount that is cell surface bound, and when additionally considering blood flow (allowing exposure to the entire vascular bed in a matter of minutes), their role is negligible. Hence, the angiotensin half-life in vivo is in the order of seconds (Danser et al., 1992), whereas in isolated plasma/serum it is easily several orders of magnitude longer (van Esch et al., 2005). Moreover, it is questionable whether the ratio at which all enzymes occur in serum or plasma is identical to that in the in vivo situation. As a consequence, incubating plasma/serum will generate ‘equilibrium levels’ (given the fact that they are obtained after an incubation period, they do not really represent levels and should, like plasma renin activity, be expressed per time component) that have little to do with the in vivo situation, and quite often metabolites occur that are not seen in vivo. Thus, the equilibrium approach might provide an insight on what enzymes are present in serum or plasma but cannot be used to draw conclusions on the in vivo levels of angiotensin metabolites.

## III. Receptors Involved in the Effects of Kidney Angiotensins

### A. AT_1_ Receptor

Ang II, Ang III, and Ang-(1-7) have all been reported to play diverse and important roles in the regulation of renal hemodynamics and tubular transport activities via activation of their respective receptors or receptor binding sites (Kobori et al., 2007b; Forrester et al., 2018; Li et al., 2018b). Ang II is the major effector of all angiotensin peptides in the kidney. Indeed, it reduces renal blood flow and determines glomerular filtration by contracting efferent arterioles more strongly than afferent arterioles. In addition, it regulates fibrosis and inflammation and stimulates sodium and bicarbonate (HCO_3_^−^) reabsorption via activation of NHE3, the basolateral Na^+^-HCO_3_^−^ cotransporter and basolateral Na^+^/K^+^-ATPase (Kobori et al., 2007b; Forrester et al., 2018; Li et al., 2018b). Finally, as has been discussed before (in section II.B), it stimulates ENaC. These well recognized effects of Ang II in the kidney are mediated primarily by AT_1_ receptors (Forrester et al., 2018; Kobori et al., 2007b), whereas the AT_2_ receptor may counteract some or all of these effects (Siragy et al., 1999; Kemp et al., 2016; Li et al., 2020). The molecular structure, pharmacological classification, and signaling transduction pathways have been well characterized and comprehensively reviewed elsewhere (Gasparo et al., 2000; Kobori et al., 2007b; Forrester et al., 2018). Signaling involves both G protein–coupled kinases and *β*-arrestins. The latter were originally believed to mediate receptor internalization and desensitization but now are also known to couple directly to MAPK, ERK1/2, and nuclear factor-kappa B (NF-*κ*B) (Violin et al., 2013). Intriguingly, this new insight has led to the development of *β*-arrestin–biased AT_1_ receptor agonists (e.g., TRV027, SII, and TRV120023), which are being evaluated in heart failure (Pang et al., 2017), based on the concept that such drugs would not only act as an ARB (reducing blood pressure, improving renal blood flow, and enhancing sodium excretion) but would also increase cardiac contractility via the *β*-arrestin pathway (Boerrigter et al., 2012; Violin et al., 2013). Yet, Wang et al. (2017c) reported that the AT_1_ receptor–*β*-arrestin–ERK1/2 signaling pathway is responsible for the development of renal fibrosis, whereas Carneiro de Morais et al. (2015) observed that TRV120023 inhibits NHE3 in opossum proximal tubule cells. Thus, whether such drugs would be useful tools in kidney disease is still unclear.

In rodents, the AT_1_ receptor is divided into two subtypes, AT_1a_ and AT_1b_ (Murphy et al., 1991), to which Ang II binds with high specificity and affinity. Interestingly, only one AT_1_ receptor gene is identified in humans. AT_1a_ receptors are coupled to Gq/11 proteins and activation of phospholipase C (Gasparo et al., 2000; Kobori et al., 2007b; Forrester et al., 2018). This leads to the generation of inositol triphosphate and diacylglycerol, with subsequent mobilization of intracellular Ca^2+^ and activation of protein kinase C. AT_1_ (AT_1a_) receptor–mediated increases in intracellular Ca^2+^ are associated with well recognized contraction of VSMC and vasoconstriction of blood vessels, whereas activation of protein kinase C triggers diverse downstream signaling transduction pathways that mediate long-term genomic and transcriptional effects such as oxidative stress responses, cellular growth, transporter expression (NHE3, Na^+^/K^+^-ATPase, and the Na^+^/HCO_3_^−^ cotransporter), tissue fibrosis, and target tissue injury (Li and Zhuo, 2011; Crowley and Rudemiller, 2017; Li et al., 2018b). The pharmacological characteristics of AT_1b_ receptors remain poorly investigated.

**Fig. 5:**
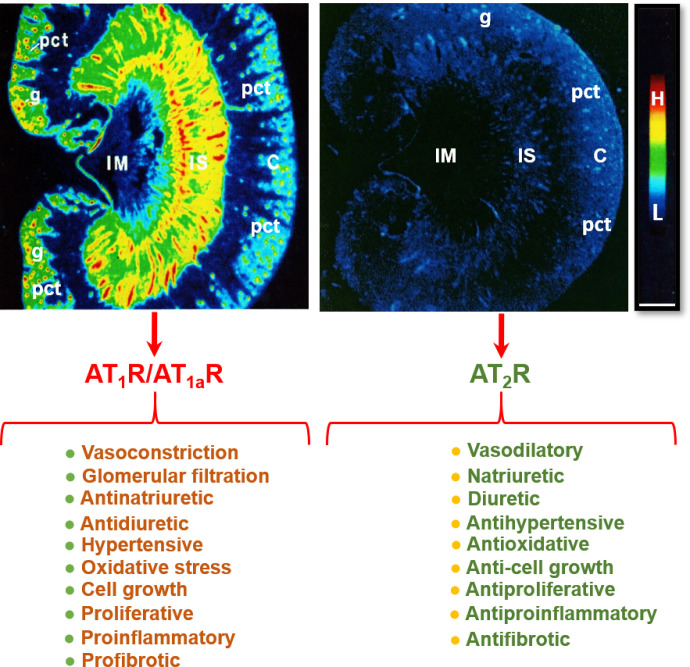
Localization of angiotensin II type 1 receptor (AT_1_R, largely representing its subtype a, AT_1a_R) and angiotensin II type 2 receptor (AT_2_R) in the rat kidney using quantitative in vitro autoradiography and opposing actions of AT_1_R/AT_1a_R and AT_2_R in the kidney. Panel A shows the anatomic localization of AT_1_R/AT_1a_R with high levels in the glomerulus (g) and the inner stripe of the outer medulla corresponding to vasa recta bundles, and moderate levels in the proximal convoluted tubules (pct) in the cortex (pct) and renomedullary interstitial cells (RMICs) in the inner stripe of the outer medulla between vasa recta bundles. The inner medulla (IM) expresses a very low level of AT_1_R/AT_1a_R. Panel B shows the anatomic localization of AT_2_R, with low levels in the outer cortex, corresponding to the glomeruli and the proximal tubules, and the inner stripe of the outer medulla, corresponding to vasa recta bundles and RMICs. Again, the IM expresses a very low level of AT_2_R. Red represents high level (H), whereas dark blue represents background levels (L). Modified from Zhuo et al. (1992, 1994).

AT_1_ receptors belong to the AT_1a_ subtype in most, if not all, target tissues, whereas AT_1b_ receptor expression is restricted to a limited number of target tissues. Thus, the majority of the effects of Ang II are mediated by AT_1a_ receptors (Gasparo et al., 2000; Kobori et al., 2007b; Forrester et al., 2018; Li et al., 2018b, 2021). Indeed, over 95% of the renal AT_1_ receptors belong to the AT_1a_ subtype, whereas AT_1b_ receptors account for only about 5% (Chen et al., 1997). The anatomic and cellular localization of AT_1_ (AT_1a_) receptors in the kidney has been well characterized using quantitative autoradiography with radiolabeled Ang II (Zhuo et al., 1992), in situ hybridization histochemistry (Aguilera et al., 1994), and immunohistochemistry (Harrison-Bernard et al., 1997). However, radioreceptor binding assays and autoradiography remains the gold-standard approach due to its highly specific ligand and receptor interactions. Indeed, we and others have used this approach to localize AT_1_ (AT_1a_) receptors in the kidneys of rat, mouse, rabbit, monkey, or humans with a striking pattern of anatomic and cellular distribution (Fig. 5) (Gibson et al., 1991; Grone et al., 1992; Sechi et al., 1992; Zhuo et al., 1992, 1993, 1996). Autoradiographs show that in the cortex, a very high density of AT_1_ receptor binding occurs in the glomerulus, where it dominates in mesangial cells and less in endothelial cells, epithelial cells, and podocytes. A moderate level of AT_1_ receptor binding is localized over the intervening outer cortex, primarily corresponding to the proximal convoluted tubules (Grone et al., 1992; Sechi et al., 1992; Zhuo et al., 1992, 1993). AT_1_ receptor binding is low to undetectable in the outer stripe of the outer medulla, where the loop of Henle is primarily located. By comparison, very high density AT_1_ receptor binding is localized in the longitudinal bands traversing the inner stripe of the outer medulla associated with the vasa recta bundles. In the interbundle area of the inner stripe of the outer medulla, there is a moderate density of AT_1_ receptor binding primarily associated with type 1 renomedullary interstitial cells (RMICs). Interestingly, the entire inner medulla (IM) of the kidney, especially toward the tip of the IM, expresses a very low level of AT_1_ receptor binding (Fig. 5). The inner medullary CDs are primarily located in this region. Consistent with the well recognized hemodynamic effect, Ang II receptor binding is also seen overlying the media of intrarenal blood vessels but with a much lower density than the binding associated with glomeruli, proximal convoluted tubules, or the vasa recta bundles.

In situ hybridization histochemistry also offers a high specificity to localize AT_1_ (AT_1a_) mRNA expression in the kidney at the light microscopic level. Due to the sensitivity of the approach, AT_1_ (AT_1a_) mRNA expression is widely seen throughout the kidney, including the blood vessels, glomerulus, proximal tubules, loop of Henle, distal tubules, and collecting ducts (Aguilera et al., 1994; Gasc et al., 1994; Healy et al., 1995). By contrast, the approach of localizing AT_1_ (AT_1a_) receptors in the kidney using immunohistochemistry with AT_1_ receptor antibodies remains highly controversial. The key issue with using immunohistochemistry to localize AT_1_ receptor proteins in the kidney or other tissues is that all commercially available AT_1_ receptor antibodies are not specific to AT_1_ receptors (Herrera et al., 2013), thus leading to false positive localization of AT_1_ receptors. Immunohistochemistry is therefore not advised to be used for AT_1_ receptor localization.

### B. AT_2_ Receptor

**Fig. 6:**
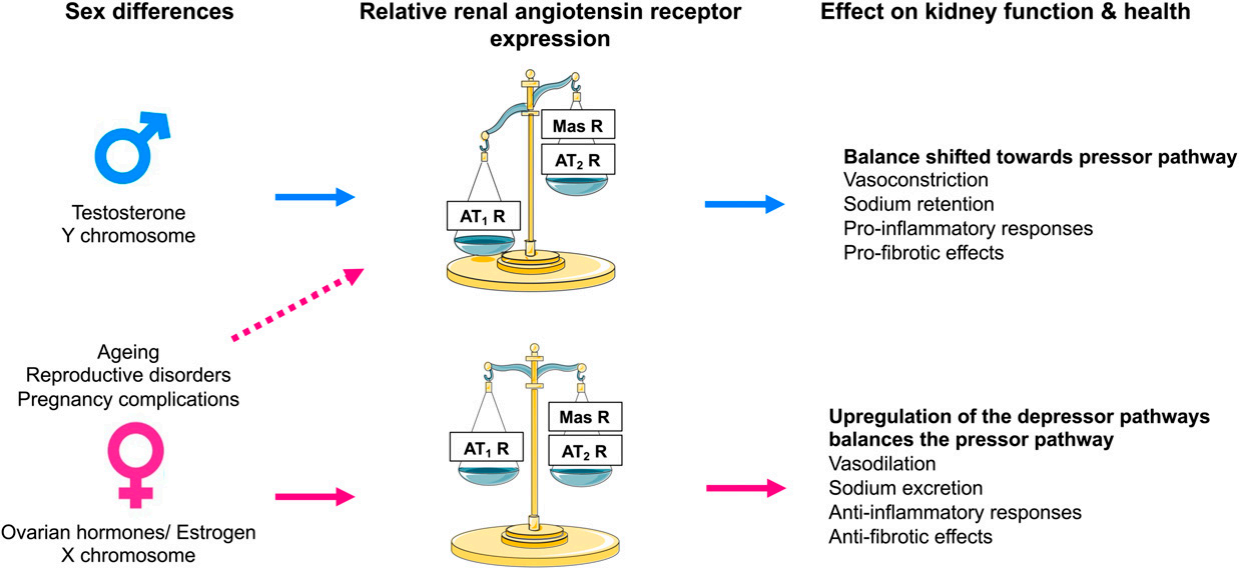
Effect of sex on renal angiotensin receptor expression and kidney function and health. Expression of the angiotensin II type 2 receptor (AT_2_R) and Mas receptor are differentially modulated by sex chromosomes and sex hormones and are influenced by female-specific conditions and diseases. Consequently, in males, the relative renal angiotensin receptor balance is skewed toward the angiotensin II type 1 receptor (AT_1_R), which enhances vasoconstriction, sodium reabsorption and proinflammatory and profibrotic effects within the kidney. Conversely, adult females have greater expression of the AT_2_ and Mas receptors than males, which counterbalances the effect of the AT_1_ receptor and enhances vasodilation, natriuresis and anti-inflammatory and antifibrotic effects within the kidney. In females, various factors such as age, menopause, and complications of pregnancy can lead to a reduction in the renal expression of AT_2_ and Mas receptors, leading to an increase in blood pressure and sodium retention and adverse effects on kidney health. MasR, Mas receptor.

The AT_2_ receptor is a seven-transmembrane G protein–coupled receptor. The AT_2_ receptor is highly expressed during fetal life (Grady et al., 1991), suggesting that it may play an important role during development. Although AT_2_ receptor expression generally declines after birth, persistent AT_2_ receptor expression can be detected in several adult tissues, including the adrenal glands, kidneys, uterus, ovaries, vasculature, heart, and brain (Matsubara et al., 1998; Sampson et al., 2012). In the adult kidney, the AT_2_ receptor is widely expressed (albeit at low levels) in the vasculature (Zhuo et al., 1996; Matsubara et al., 1998), glomeruli, and tubular segments (Miyata et al., 1999; Bosnyak et al., 2010). Stimulation of the AT_2_ receptor generally opposes the classic effects of AT_1_ receptor stimulation by inducing vasodilation, natriuresis, and antifibrotic and anti-inflammatory effects (Fig. 5). The vasodilatory effects of the AT_2_ receptor are mediated via an increase in the production of nitric oxide (NO) and cGMP, which is achieved either by increasing bradykinin production with a subsequent effect mediated through bradykinin type 2 receptors or directly via activation of NO production independent of bradykinin (Padia and Carey, 2013). However, there are reports of AT_2_ receptor–mediated vasoconstriction as well as no effect of AT_2_ receptor stimulation on vascular tone, as reviewed previously (Verdonk et al., 2012a). Similarly, there are numerous studies that have reported no effect of AT_2_ receptor stimulation or blockade on renal function (Uhlenius et al., 2002; Chappellaz and Smith, 2007; Welch et al., 2007). The discrepancy between these findings may be related, at least in part, to the specificity of the tools used to interrogate the function of the AT_2_ receptor and the relative AT_2_:AT_1_ receptor ratio, which is integral in determining the effects of RAS stimulation on vascular tone and kidney function. For example, the nonpeptide AT_2_ receptor agonist compound 21 (C21), which is 4000-fold more selective for the AT_2_ receptor than the AT_1_ receptor, is not specific for the AT_2_ receptor at higher doses (i.e., within the micromolar range) (Verdonk et al., 2012b). As discussed below, accumulating evidence suggests that renal AT_2_ receptor expression and function is greater in females than in males (Fig. 6). Of note, recent studies utilizing RNA sequencing to profile the entire length of the nephron (performed almost exclusively using tissues obtained from adult male rodents) have demonstrated that expression of the AT_2_ receptor is low to negligible (Chen et al., 2021). This may explain why the effects of AT_2_ receptor stimulation are often only unmasked in the presence of an ARB (Padia and Carey, 2013) and that recent studies investigating the natriuretic effects of the AT_2_ receptor have been performed largely in female rodents.

Targeted disruption of the mouse *Agtr2* gene has demonstrated that AT_2_ receptor deficiency results in a rightward shift of the pressure-natriuresis relationship (Gross et al., 2000), increased salt-sensitivity (Siragy et al., 1999), and enhanced sensitivity to Ang II infusion, which is associated with reduced urinary sodium excretion (Siragy et al., 1999) and bradykinin and cGMP levels (Gross et al., 2000). In rats, both systemic and intrarenal infusion of C21 has been demonstrated to induce natriuresis, and this effect was completely abolished by concomitant intrarenal infusion of the AT_2_ receptor antagonist PD123319 (Hilliard et al., 2012; Kemp et al., 2016). Since AT_2_ receptor activation increases renal blood flow but not GFR (Hilliard et al., 2012, 2014; Kemp et al., 2014, 2016), AT_2_ receptor–mediated natriuresis must be due to changes in tubular function rather than renal hemodynamics. Within the proximal tubule, AT_2_ receptor–mediated natriuresis is achieved by translocation of AT_2_ receptors to the apical plasma membrane of proximal tubular cells (Kemp et al., 2014, 2016; Krause et al., 2020), inhibition of HCO_3_^−^ reabsorption (Haithcock et al., 1999), and the translocation, internalization, and inactivation of NHE3 in the apical membrane and Na^+^/K^+^-ATPase in the basolateral membrane (Kemp et al., 2014). The latter occurs via the NO/cGMP pathway. The AT_2_ receptor also decreases AT_1_ receptor expression and function via the NO/cGMP/specificity protein 1 serine phosphorylation pathway (Yang et al., 2012a). Additionally, activation of the AT_2_ receptor reduces renal fibrosis either directly or indirectly via its anti-inflammatory effects and stimulation of the NO/cGMP pathway. In the 2-kidney, 1-clip hypertension model (Matavelli et al., 2011) and stroke-prone SHRs (Rehman et al., 2012), AT_2_ receptor agonism with C21 reduced renal inflammation and fibrosis, which was associated with improved production of NO and cGMP.

Accumulating evidence suggests that Ang III is the endogenous intrarenal ligand for the AT_2_ receptor. Studies in normotensive rat kidneys have demonstrated that Ang III, but not Ang II, induced AT_2_ receptor–mediated natriuresis (Padia et al., 2007) and that this effect was mediated via activation of proximal tubule AT_2_ receptors via a cGMP-dependent pathway (Kemp et al., 2012). Similar findings have been reported recently in response to a novel *β*-amino acid substituted Ang III, *β*-Pro^7^-Ang III, which has high specificity for the AT_2_ receptor (Krause et al., 2020). Further, the Ang III–induced AT_2_ receptor–mediated natriuretic effect was augmented by the blockade of aminopeptidase N, an enzyme metabolizing Ang III to Ang IV, thereby increasing Ang III levels (Padia et al., 2007; Kemp et al., 2012). Interestingly, Ang III–induced AT_2_ receptor–mediated natriuresis is not observed in SHRs (Padia et al., 2009), indicating that a deficit in AT_2_ receptor–mediated natriuresis contributes to the development of hypertension in this model.

The location of the *Agtr2* gene on the X chromosome suggests that the AT_2_ receptor may have sexually dimorphic actions. Using the four-core genotype model, where the *Sry* gene is translocated to chromosome 3, making it possible to differentiate between effects that are sex hormone and/or sex chromosome complement dependent (Pessôa et al., 2015), it has been demonstrated that the X chromosome increases renal AT_2_ receptor expression (Dadam et al., 2017). Furthermore, numerous animal studies have demonstrated that renal AT_2_ receptor expression is greater in females than in males (Hilliard et al., 2014; Mirabito et al., 2014) and that this effect is linked to either estrogen, ovarian hormones, or reproductive status (Mirabito et al., 2014; Barsha et al., 2021). Thus, the AT_2_ receptor may contribute to the relative cardiorenal protection observed in adult females compared with age-matched males and reproductively senescent (e.g., postmenopausal) females.

Studies in rodents provide strong evidence of an enhanced functional role for renal AT_2_ receptors in females (Fig. 6). For example, genetic AT_2_ receptor deficiency resulted in a rightward shift of the chronic pressure-natriuresis relationship in adult female mice compared with their wild-type and age-matched male counterparts (Mirabito et al., 2014). In anesthetized rats, acute pharmacological stimulation of the AT_2_ receptor with graded doses of C21 (Hilliard et al., 2012) and the highest dose of *β*-Pro^7^-Ang III tested (Krause et al., 2020) produced a greater renal vasodilator response in normotensive females than in males. Moreover, in female rats, the vasodilatory and natriuretic effects induced by C21 were of a slightly greater magnitude in normotensive rats than in SHRs. These responses to C21 treatment were completely absent in male SHRs (Hilliard et al., 2014). In mice, Ang II–induced contraction in interlobar arteries was significantly attenuated in females compared with males via an AT_2_ receptor–mediated NO-dependent pathway (Viegas et al., 2012). Hilliard et al. (2011) have shown that at low renal perfusion pressures in female rats, the AT_2_ receptor maintains autoregulation of renal blood flow and GFR. Furthermore, the sensitivity of the tubuloglomerular feedback mechanism to Ang II is reduced by the presence of the AT_2_ receptor in female but not male mice (Brown et al., 2012). There is evidence to suggest that the enhanced functional role of renal AT_2_ receptors in females diminishes with age. In association with a reduction in renal AT_2_ receptor expression, the chronic pressure-natriuresis relationship is shifted rightward (Mirabito et al., 2014) and the hypertensive response to Ang II is enhanced (Barsha et al., 2021) in aged reproductively senescent females compared with their adult counterparts. In these studies, genetic or pharmacological AT_2_ receptor deficiency did not alter the response in the aged females. Excitingly, in aged females, estrogen replacement reduced the pressor response to Ang II to that observed in adult females and this effect was associated with an upregulation in renal AT_2_ receptor expression (Barsha et al., 2021), suggesting that estrogen replacement may restore the effects of the AT_2_ receptor on kidney function in aged females.

Although sex differences in AT_2_ receptor expression and function have not been examined in humans, there is evidence to suggest a greater functional role for the AT_2_ receptor in women than in men. For example, in healthy adults the renal pressor response to Ang II was blunted in women compared with men (Miller et al., 2006), and in response to treatment with an ARB, Ang II sensitivity is decreased to a greater extent in women than in men (Hudson et al., 2007). Finally, renal APA levels are greater in women than in men (Martínez et al., 1998), suggesting higher levels of Ang III and an enhanced AT_2_ receptor–mediated natriuretic response in women.

Of interest, expression of the AT_2_ receptor can be upregulated during pathologic settings such as myocardial infarction and kidney damage (Oishi et al., 2003; Vázquez et al., 2005), which has led to the notion that AT_2_ receptor agonism may be a novel therapeutic option for cardiovascular disease. Yet, preclinical studies indicate that AT_2_ receptor agonists are not suitable as a monotherapy for cardiovascular disease and that at best, AT_2_ receptor agonists may be beneficial in combination with existing RAS inhibitors (Danyel et al., 2013). Currently, the clinical focus of AT_2_ receptor agonists (in development by Vicore Pharma) is severe lung disorders, with C21 receiving orphan drug status for idiopathic pulmonary fibrosis by the US Food and Drug Administration and the European Medicines Agency and a post hoc analysis of a recent phase 2 clinical trial, suggesting that 7-day treatment with C21 in hospitalized patients with COVID-19 reduced the requirement for oxygen at day 14 (Tornling et al., 2021).

### C. Mas Receptor

The Mas receptor was first described in 1986 as Mas oncogene (Young et al., 1986), and it was not until 2003 that the Mas receptor was identified as a G protein–coupled receptor with high affinity for Ang-(1-7) (Santos et al., 2003). Within the kidney, the Mas receptor is expressed in afferent arterioles and the apical surface of tubular epithelium (Alenina et al., 2008). Upon stimulation with Ang-(1-7), the Mas receptor undergoes endocytosis (Gironacci et al., 2011) and is then slowly recycled back to the plasma membrane (Cerniello et al., 2017). Similar to the AT_2_ receptor, stimulation of the Mas receptor generally opposes the effects of the AT_1_ receptor stimulation by inducing vasodilation, natriuresis, and antifibrotic and anti-inflammatory effects. In the vasculature, Ang-(1-7)/Mas receptor stimulation increases NO release by inducing protein kinase B phosphorylation and the activation of endothelial NO synthase and inhibits endothelial Ang II–induced ROS production, leading to improved endothelial function (Sampaio et al., 2007a,b). Further, Ang-(1-7) has been demonstrated to potentiate bradykinin-induced vasodilation in various models, including porcine coronary arteries, canine coronary rings, and bovine aortic endothelial cells, an effect that is diminished by nitric oxide synthase inhibition and the bradykinin type 2 receptor antagonist HOE 140 (Li et al., 1997; Heitsch et al., 2001; Tom et al., 2001). The Mas receptor also hetero-oligomerizes with the AT_1_ receptor to act as a physiologic antagonist of the AT_1_ receptor (Kostenis et al., 2005). Consequently, Mas receptor–deficient mice have impaired endothelial function and higher blood pressure than their wild-type counterparts (Xu et al., 2008). Within the kidney, Ang-(1-7) alters sodium and HCO_3_^−^ reabsorption and stimulates the release of NO, PGE2, and prostaglandin I2 (PGI2) and augments the vasodilator effects of bradykinin (Chappell, 2012). Here it should be noted that the renal salt-handling response to Ang-(1-7)/Mas receptor stimulation (i.e., whether it will induce natriuresis or antinatriuresis) depends on a number of factors, including water and electrolyte balance, species, sex, pregnancy, nephron segment, and disease. Nevertheless, the ACE2/Ang-(1-7)/Mas receptor axis and the AT_2_ receptor are often collectively referred to as the protective RAS pathways.

The majority of studies have demonstrated that Ang-(1-7) exerts natriuretic and diuretic effects via activation of the Mas receptor (DelliPizzi et al., 1994; Santos et al., 1996). Within the proximal tubule, Mas receptors alter sodium, HCO_3_^−^, and fluid reabsorption via NHE3 (Castelo-Branco et al., 2013). Ang-(1-7) also inhibits sodium transport in the thick ascending loop of Henle via Mas receptor–dependent increases in NO (Dibo et al., 2019). Accumulating evidence also indicates that AT_1_ and AT_2_ receptors contribute to the natriuretic and diuretic effects of Ang-(1-7) (Patel et al., 2017), although the exact mechanisms remain unclear. For example, studies suggest that the Mas receptor can interact with the AT_1_ receptor and prevent its activation either by acting directly at the AT_1_ receptor (Galandrin et al., 2016) or indirectly by heterodimerizing with the AT_1_ receptor to act as a physiologic antagonist (Kostenis et al., 2005). Conversely, in certain settings, including in normotensive and hypertensive rats (Simões e Silva et al., 1998), after an acute water load (Santos et al., 1996), and in virgin females (Joyner et al., 2008), Ang-(1-7) can induce antidiuresis. This effect appears to be independent of vasopressin (Santos and Baracho, 1992) and is instead mediated via effects of Ang-(1-7) on proximal tubules (Garcia and Garvin, 1994) and inner medullary collecting ducts (Santos et al., 1996). In addition to the tubular effects of Ang-(1-7)/Mas receptor stimulation, studies in normotensive and hypertensive rats have demonstrated that renal blood flow is also modulated by Ang-(1-7) and that this effect is abolished by blockade of the Mas receptor or inhibition of prostaglandin release and NO, which are thought to be downstream of Mas receptor activation (Sampaio et al., 2007b).

Compared with wild-type mice, *Mas receptor* KO mice have decreased urine volume and natriuresis, similar free water clearance, and increased inulin clearance and microalbuminuria concomitant with a reduced renal blood flow, suggesting hyperfiltration (Pinheiro et al., 2009). Histologic examination of the kidneys of these mice indicates that Mas receptor deficiency impairs the glomerular filtration barrier, as evidenced by a reduction in the glomerular tuft diameter, and promotes a profibrotic phenotype with increased expression of fibronectin and collagens III and IV (Pinheiro et al., 2009). Importantly, these changes were associated with increased renal mRNA expression of the AT_1_ receptor and TGF*β*1 (Pinheiro et al., 2009). Similarly, in models of obstructive and DKD, it has been demonstrated that Ang-(1-7) reduced TGF*β*1 expression and signaling through TGF*β*1/Smad complex and that these effects were reversed by Mas receptor blockade (Liu et al., 2012). Ang-(1-7)/Mas receptor also modulates MAPKs, which may contribute to its antioxidant, anti-inflammatory, and antifibrotic effects. For example, in the presence of Ang II or high glucose in proximal tubule cells, the binding of Ang-(1-7) to the Mas receptor inhibits phosphorylation of MAPKs, including p38, ERK1/2, and c-Jun N-terminal kinase (Su et al., 2006; Gava et al., 2009). Conversely, in dual *ACE2* and *Mas receptor*–deficient mice, Ang II–induced hypertension resulted in severe hypertensive nephropathy, which was associated with decreased creatinine clearance and increased renal fibrosis, inflammation, and AT_1_ receptor-ERK1/2-Smad3 and NF-*κ*B signaling (Ni et al., 2020). Collectively, these findings support a key role for the ACE2/Ang-(1-7)/Mas receptor axis in the maintenance of kidney health. Similar to the AT_2_ receptor, evidence suggests a sex-specific role for the ACE2/Ang-(1-7)/Mas receptor axis. Renal mRNA expression of ACE2 and Mas receptor and renal cortical Ang-(1-7) levels are greater in female versus male rodents (Sullivan et al., 2010; Sampson et al., 2012; Mirabito et al., 2014; Zimmerman et al., 2015). Moreover, in both normotensive and hypertensive rats, it has been reported that exogenous Ang II infusion induces a greater increase in renal cortical Ang-(1-7) levels in females than in males (Sullivan et al., 2010; Zimmerman et al., 2015). Interestingly, Mas receptor expression was increased in the renal cortex after Ang II infusion in females only (Sullivan et al., 2010), suggesting that Ang-(1-7) elicits its effects via the AT_2_ receptor in adult males. Consistent with this notion, it has been demonstrated that Ang-(1-7) elicits a vasodepressor response in adult males, which is blocked by coadministration of the AT_2_ receptor antagonist PD123319 (Bosnyak et al., 2012). Further, in the presence of Mas receptor blockade, renal blood flow decreased significantly in anesthetized female but not male normotensive rats (Safari et al., 2012).

In contrast to the studies that suggest a protective role for the ACE2/Ang-(1-7)/Mas receptor axis, accumulating evidence suggests that downregulation of this pathway may be beneficial in disease settings. For example, genetic and pharmacological Mas receptor deficiency has been reported to prevent high-fat diet–induced kidney injury (Kong et al., 2021), abolish salt-sensitive hypertension (Heringer-Walther et al., 2012), reduce kidney damage in models of kidney injury (e.g., unilateral ureteral obstruction and ischemia/reperfusion injury) (Esteban et al., 2009), and inhibit NF-*κ*B activation and thus the upregulation of proinflammatory cytokines (Esteban et al., 2009). Clearly, more studies are needed to establish the contribution of the ACE2/Ang-(1-7)/Mas receptor axis in kidney health and disease, also in view of the fact that recent studies do not support that Ang-(1-7) is the endogenous agonist of the Mas receptor (Gaidarov et al., 2018). Moreover, renal Ang-(1-7) levels, if detectable at all, are usually far below those of Ang II (Campbell et al., 1995a; Roksnoer et al., 2015; Bovée et al., 2021; Uijl et al., 2021). This raises the question of whether Ang-(1-7)-Mas receptor interaction truly occurs under physiologic conditions.

### D. Localization and Role of Intracellular Angiotensin Receptors in the Kidney

The renal effects of Ang II mediated by cell surface receptors have been extensively studied (Kobori et al., 2007b). However, whether Ang II receptors are present intracellularly, and if so, where they are localized in the cell, and whether intracellular Ang II receptors mediate any biologic or physiologic effects of intracellular Ang II remains poorly understood. The concept of an intracrine or intracellular Ang II system is not new. Initially, two possibilities emerged to explain the ‘local’ effects of Ang II. First, that Ang II is generated intracellularly and then released into the extracellular fluid compartment to induce effects via cell membrane receptors in an autocrine or paracrine manner. Second, that Ang II is generated extracellularly, on the cell surface by membrane-bound ACE, and then acts on neighboring membrane receptors. Of course, after intracellular expression, AT receptors will appear on the cell membrane, whereas after Ang II binding, cell membrane AT_1_ receptors are expected to internalize (potentially resulting in desensitization), with internalized Ang II subsequently being degraded in the lysosomal pathway. As discussed earlier, the second possibility now is the most likely, given that renal intracellular Ang II disappeared in animals that lack all AT receptors (van Kats et al., 2001b; van Esch et al., 2010b), whereas conceptually it is hard to envision a combination of either internalization or intracellular expression of renin, AGT, and ACE, allowing them to perform their reaction cascade in the same intracellular compartment.

However, there is accumulating evidence supporting the presence of intracellular Ang II that plays important roles in diverse biologic and physiologic responses (Cook et al., 2001; Zhuo et al., 2006; Li and Zhuo, 2008; Li et al., 2011, 2020). This Ang II may simply represent internalized Ang II of extracellular origin. In the rat liver, early studies have reported that Ang II receptor binding sites are present in isolated nuclei in which Ang II directly induced effects, including AGT transcription (Eggena et al., 1996). In the heart, administration via intracellular dialysis of renin, AGT, or Ang II directly into hamster cardiomyocytes was demonstrated to alter intercellular communications (De Mello, 1998), whereas in VSMCs, microinjection of Ang II directly into the cells elicited intracellular and nuclear calcium responses (Haller et al., 1996). As these intracellular responses to intracellular Ang II administration were blocked by concurrent intracellular administration of ARBs, these early studies support the proof-of-concept hypothesis that intracellular AT_1_ receptors mediated these effects. More recent studies on intracellular Ang II and Ang II receptors involved the development and application of an intracellular cyan fluorescent Ang II fusion protein, ECFP/Ang II (Cook et al., 2001; Li et al., 2011, 2020). This Ang II fusion protein is constructed to be expressed intracellularly without being released into the extracellular fluid compartment, and it only acts on intracellular AT receptors to induce intracellular effects.

**Fig. 7:**
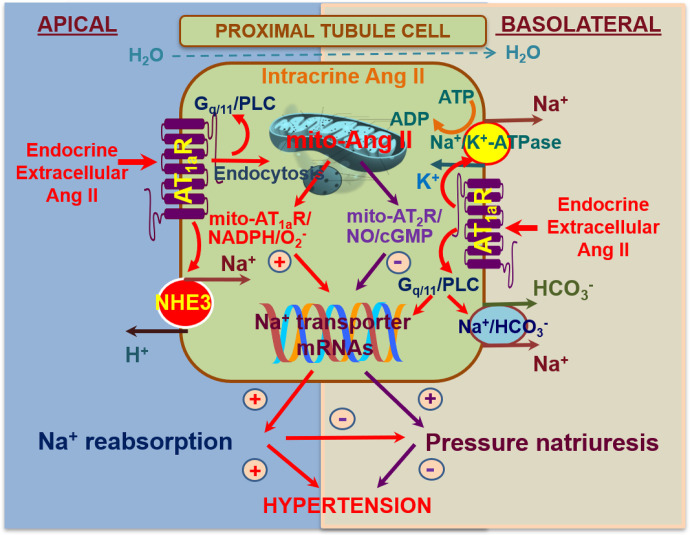
Scheme showing that extracellular (endocrine and paracrine) angiotensin (Ang) II is taken up via Ang II type 1a receptor (AT_1a_R)-mediated internalization and then results in G_q/11_/phospholipase C (PLC) signaling. Under physiologic conditions (low Ang II), internalized Ang II is sorted to the lysosomal pathway for degradation, whereas AT_1a_ receptors recycle back to the membrane. Alternatively, particular at sustained high extracellular Ang II levels, the Ang II–AT_1a_R complex may bypass the lysosomal degradation pathway, allowing its transport to mitochondria and nucleus, where Ang II activates AT_1a_R and/or Ang II type 2 receptor (AT_2_R) to alter mitochondrial oxidative and glycolysis stress responses. This may in turn alter the expression or activity of Na^+^/H^+^ exchanger 3 (NHE3) on the apical membranes, or Na^+^/K^+^-ATPase and the Na^+^/HCO_3_^−^ cotransporter on the basolateral membranes in the proximal tubules. Thus, activation of the mitochondrial Ang II/AT_1a_R/O_2_^−^ signaling will stimulate proximal tubule sodium reabsorption, impair the pressure-natriuresis response, and elevate blood pressure. Conversely, activation of the mitochondrial Ang II/AT_2_R/NO/cGMP signaling by overexpressing AT_2_R in the mitochondria will likely inhibit proximal tubule sodium reabsorption, augment the pressure-natriuresis response, and lower blood pressure. Modified from Li et al. (2020, 2021).

In the kidney, intracellular AT_1_ (AT_1a_) and AT_2_ receptors have been reported in freshly isolated endosomes (Zhuo et al., 2002), endoplasmic reticulum (Ferrão et al., 2017), mitochondria (Li et al., 2020), and nuclei from the renal cortex (Li and Zhuo, 2008). Most of these intracellular AT receptors in the renal cortex are associated primarily with the proximal tubules of the kidney and are likely derived from both intracellular expression and Ang II–induced internalization and subsequently trafficking to endoplasmic reticulum, mitochondria, and nucleus. Whether intracellular AT receptors are localized in other renal cells has not been studied. In contrast to the mainstream views, not all internalized Ang II and AT_1_ (AT_1a_) receptors are sorted into the lysosomal degradation pathways, especially in a chronic and high Ang II environment in the in vivo setting (van Kats et al., 1997, 2001b; Zhuo et al., 2002; Li et al., 2007, 2009; Wilson et al., 2016). Some internalized Ang II and AT_1_ (AT_1a_) receptors bypass the lysosomal degradation pathways and are transported to other intracellular organelles. Thus, internalized Ang II may serve as an important source of intracellular Ang II (Fig. 7), which may stimulate its cytoplasmic and nuclear AT_1_ or AT_2_ receptors to induce important biologic and physiologic effects.

This hypothesis has been tested in both cultured proximal tubule cells and the proximal tubules of the kidney, and the data support an important biologic and physiologic role of intracellular Ang II via acting on intracellular, mitochondrial, and nuclear AT_1_ (AT_1a_) and AT_2_ receptors (Zhuo et al., 2006; Li and Zhuo, 2008; 2013; Li et al., 2011, 2020, 2021). Early in vitro studies demonstrated that microinjection of Ang II directly into cultured rabbit proximal tubule cells significantly increased intracellular Ca^2+^ responses, even during blockade of cell surface AT_1_ receptors with losartan (Zhuo et al., 2006). Since concurrent microinjection of losartan prevented the Ca^2+^ response, it is likely induced via activation of intracellular AT_1_ receptors (Haller et al., 1996; Zhuo et al., 2006). In freshly isolated rat renal cortical nuclei, incubation with Ang II significantly increased transcriptional responses on TGF*β*1, monocyte chemoattractant protein-1, and NHE3 mRNA expression (Li and Zhuo, 2008). Since freshly isolated nuclei were completely devoid of endosomes, lysosomes, endoplasmic reticulum, mitochondria, and cell membranes, these Ang II–induced transcriptional responses must have been due to direct actions of Ang II via activation of nuclear AT receptors (Li and Zhuo, 2008). These in vitro effects of intracellular Ang II were further supported by in vivo animal studies in which an intracellular Ang II fusion protein was expressed selectively in the proximal tubules of the kidney (Li et al., 2011, 2020, 2021). Adenovirus-mediated overexpression of the intracellular Ang II fusion protein selectively in the mitochondria of the proximal tubule cells induced two key mitochondrial functional responses: oxygen consumption rate and extracellular acidification rate (Li et al., 2020). Finally, overexpression of this Ang II fusion protein in the mitochondria of the proximal tubules in mice increased blood pressure, and this effect was attenuated in mutant mouse models with proximal tubule–selective deletion of either AT_1a_ receptors or NHE3 (Li et al., 2020, 2021). This suggests that intracellular Ang II in the mitochondria of the proximal tubules may play an important role in the physiologic regulation of proximal tubule sodium reabsorption and blood pressure homeostasis.

## IV. Intrarenal RAS and Disease

### A. Hypertension

It is widely believed that the intrarenal RAS plays a central role in the development and maintenance of hypertension. Here the acute effects of Ang II on salt and water retention are obvious players, particularly when combined with the long-term effects of Ang II on growth, remodeling, and inflammation. The activation of the renal RAS does not necessarily run in parallel with that of the systemic RAS (Navar et al., 2002; Kobori et al., 2007b). Initially this was attributed to upregulated local expression of AGT in the proximal tubule (e.g., in response to systemic Ang II infusion). Although kidney-specific AGT overexpression in transgenic mice indeed resulted in hypertension (Davisson et al., 1999), we now know, as discussed above in section II.C, that the role of locally synthesized AGT in the kidney is marginal, if not absent. A more likely scenario is that high blood pressure (e.g., after infusing Ang II) affects glomerular filtration, thus resulting in the accumulation of filtered hepatic AGT into the kidney and allowing increased Ang II generation (Koizumi et al., 2019). Since hypertension will normally suppress renin release (Danser et al., 1998), it is not unlikely that low systemic RAS activity indeed occurs in combination with high renal RAS activity (due to enhanced AGT uptake).

Given that both circulating renin and renin at renal tissue sites derive from JG cells, it is difficult to conceive a situation where local renin release is upregulated while systemic release is down. It is for this reason that CD (pro)renin production seemed an attractive concept. As discussed in section II.B, chronic Ang II infusion would then upregulate CD (pro)renin (Prieto-Carrasquero et al., 2004) and simultaneously suppress JG renin synthesis, thus again resulting in the combination of low systemic renin and high renal/CD RAS activity, with the latter potentially involving the (P)RR. However, given the absence of renin lineage cells at the level of the CD (Tang et al., 2019), local (pro)renin synthesis at this location seems impossible. An alternative explanation would be that CD (pro)renin represents enhanced filtered circulating (pro)renin, particularly since Ang II not only impairs the glomerular filtration barrier but also downregulates megalin, thereby preventing (pro)renin reabsorption in the proximal tubule (see section II.B).

Renal ACE is another contributor to hypertension, and various hypertension models support its upregulation under hypertensive conditions (Gonzalez-Villalobos et al., 2013; Giani et al., 2014). Here an obvious question is whether this is the cause or consequence of hypertension. Mice expressing ACE exclusively in the kidney tubules but not in other tissues displayed hypertension when infused with Ang I (Gonzalez-Villalobos et al., 2011). Yet, mice devoid of renal ACE were resistant to hypertension induced by Ang II infusion or the NO synthase inhibitor N^G^-nitro-L-arginine methyl ester (L-NAME) (Gonzalez-Villalobos et al., 2013; Giani et al., 2014). The authors argued that both Ang II infusion and L-NAME treatment upregulated renal ACE 1.5- to 2-fold, thus enhancing local Ang II generation and sodium transporter activation and subsequently leading to hypertension via a reduction in natriuresis. The same authors showed that mice specifically expressing ACE in renal tubular epithelium developed salt-sensitive hypertension in response to L-NAME, whereas mice selectively lacking renal tubular epithelial ACE did not. Thus, it is particularly Ang II generated by tubular epithelial ACE that contributes to salt-sensitive hypertension (Giani et al., 2017). A caveat of these studies is that renal ACE in the mice that were supposed to lack renal ACE was not zero but rather in the order of 10%–15% of wild-type levels. This resembles what can be achieved during ACE inhibition (see section V.A) and raises the question of what the renin levels were in these models. Normally, such a reduction of ACE activity can be easily matched by 5- to 10-fold rise in renin. Thus, before definitely invoking a modest rise in renal ACE as the underlying cause of the blood pressure rise, future studies should rule out that it is not simply due to changes in renin.

Crowley et al. (2005, 2006) used a kidney crosstransplantation strategy to generate mice expressing AT_1_ receptors either only within or only outside the kidney, thus demonstrating that activation of renal AT_1_ receptors induced Ang II–dependent hypertension whereas exclusive extrarenal AT_1_ receptor stimulation was insufficient to cause hypertension. These results support a pivotal role of renal AT_1_ receptors in blood pressure regulation. Moreover, proximal tubule–specific AT_1_ receptor ablation lowered blood pressure and prevented Ang II–induced hypertension by reducing sodium and fluid reabsorption (Gurley et al., 2011; Li et al., 2021).

Taken together, changes in renal AT_1_ receptor density combined with alterations in local ACE and the reabsorption of filtered (pro)renin and AGT allow an upregulation of Ang II–mediated effects in the kidney, even in the absence of changes in the circulating RAS or when circulating RAS activity is low. These local effects of Ang II may contribute to both the development and maintenance of hypertension, and this explains why RAS blockers are also effective in patients with low to normal circulating RAS activity.

### B. Chronic Kidney Disease

The renoprotective effects of RAS inhibitors beyond blood pressure control have been recognized since the 1990s. ARBs and ACEIs have been the first line of therapy for hypertensive chronic kidney disease (CKD) ever since (Jafar et al., 2001), especially in patients with albuminuria or DKD (Palmer et al., 2015). A prevailing hypothesis is that the initial kidney injury activates the intrarenal RAS causing local Ang II production, thereby driving CKD progression. This is not surprising since Ang II not only causes vasoconstriction and increases glomerular pressure and sodium retention but, in addition to these glomerular and tubular effects, also induces inflammation, fibrosis, and glomerular sclerosis, thereby leading to further nephron loss (Rüster and Wolf, 2006). To address the role of the intrarenal RAS, most studies have relied on measuring RAS components in kidney tissue or urine. Here it is difficult to distinguish the circulating and renal RAS, except in the case of ACE and AT receptors.

An increase in renal RAS components has been observed in several animal models of CKD. For example, in models of kidney mass reduction, tubular renin and Ang II are increased and early RAS inhibition prevents a further GFR decline (Gilbert et al., 1999). Animals who are made diabetic with STZ generally show an increase in renal Ang II and urinary AGT prior to a decrease in GFR and the development of albuminuria (Singh et al., 2005; Kamiyama et al., 2012). In other diabetic animal models, including Zucker rats (a genetic obesity animal model caused by mutation in the leptin receptor), Otsuka Long-Evans Tokushima Fatty rats, and db/db mice, kidney Ang II and AGT are increased both before and after the development of kidney damage (Nagai et al., 2005; Miyata et al., 2008), although not all investigators agree (Leehey et al., 2008). Finally, in rodent models of glomerular disease, including IgA nephropathy and antithymocyte serum nephritis, an increase in kidney RAS components compared with healthy controls was observed (Ohashi et al., 2008; Huang et al., 2012).

Several groups have reported that RAS component expression in human kidney biopsies correlates with the presence and severity of the underlying kidney disease (Lai et al., 1998; Del Prete et al., 2003; Mezzano et al., 2003; Konoshita et al., 2006; Kobori et al., 2007a; Takamatsu et al., 2008; Ohashi et al., 2017; Zhang et al., 2019). In patients with mixed etiology kidney disease, RAS component mRNA levels were upregulated in glomeruli and tubules compared with controls (Lai et al., 1998). Renal tissue AGT and AT_1_ receptor density associated with the degree of fibrosis in patients with kidney failure (Ohashi et al., 2017). In kidney biopsies taken from patients with DKD and IgA nephropathy, higher RAS abundance has been consistently observed. Patients with DKD show increased RAS abundance in tubular and interstitial cells compared with healthy controls (Mezzano et al., 2003) and hence respond less strongly to RAS blockade (Price et al., 1999; Lansang et al., 2001). Kidney biopsies from patients with DKD also show increased ACE mRNA compared with patients with non-DKD CKD (Konoshita et al., 2006). In patients with IgA nephropathy, an overexpression in RAS genes has been observed (Del Prete et al., 2003). IgA nephropathy increased AGT and Ang II immunoreactivity along the tubule, and AGT was present in glomeruli (Kobori et al., 2007a). Greater AGT and Ang II immunoreactivity was also observed in glomeruli from pediatric patients with IgA nephropathy (Takamatsu et al., 2008). Expression of the AT_1_ receptor in patients with IgA nephropathy is related to disease severity and reduced by RAS inhibition (Zhang et al., 2019).

Although generally the circulating RAS is up in CKD, it is not in patients with diabetes, whose plasma renin levels are low (Price et al., 1999). This has been known for decades, and simultaneously the remarkable observation was made that circulating prorenin in patients with diabetes is elevated (Luetscher et al., 1985). Since prorenin levels in patients with diabetes correlated with microvascular complications, including nephropathy and retinopathy, initial studies focused on the eye as a potential source of this prorenin. Although this led to the discovery that prorenin is indeed made in the eye, its relatively low ocular levels, combined with the low ocular flow, made the release of significant quantities of prorenin from the eye into the blood stream unlikely (Danser et al., 1989). Next, the concept of the CD as the source of this prorenin, particularly in combination with the (P)RR as a prorenin activator, caused excitement (Kang et al., 2008). However, as discussed in section II.B, this concept is now also being abandoned, leaving the possibility that the elevated prorenin levels in diabetes originate in the JG cells after all. Whether this prorenin underlies the increased renal RAS activity in diabetes remains unknown. This increased renal RAS activity is in agreement with the overwhelming clinical evidence that RAS blockers are beneficial in diabetes (Lewis et al., 1993; Haller et al., 2011), most likely in a blood pressure–independent manner (Roksnoer et al., 2016b).

As the majority of patients with CKD are not biopsied, urinary AGT excretion has frequently been used as proxy for intrarenal RAS activity (Table 3). Most (Yamamoto et al., 2007; Nishiyama et al., 2011; Jang et al., 2012; Zhang et al., 2013; Urushihara et al., 2015) but not all (Jang et al., 2014) studies that did compare AGT excretion with biopsy data showed a correlation between kidney Ang II abundance and urinary AGT excretion (often measured in spot urine as urinary AGT to creatinine ratio) (Yamamoto et al., 2007; Nishiyama et al., 2011; Jang et al., 2012; Zhang et al., 2013; Urushihara et al., 2015). In addition, several studies identified a relationship between histologic damage or fibrosis and urinary AGT excretion (Yamamoto et al., 2007; Kim et al., 2011a; Jang et al., 2012; Urushihara et al., 2015). Urinary AGT excretion is the net effect of glomerular filtration, tubular reabsorption, and possibly local release along the nephron (see section II.C). Kidney disease may impact all of these processes. Damage to the glomerular filtration barrier is common in CKD and will increase AGT filtration (Matsusaka et al., 2014; Koizumi et al., 2019). Damage to the proximal tubule or changes in megalin function will alter AGT reabsorption, and impaired AGT reabsorption has been observed in DKD (Tang et al., 2019) and inherited kidney diseases such as Dent’s disease (Roksnoer et al., 2016a) and autosomal dominant polycystic kidney disease (ADPKD) (Salih et al., 2017). Differences in AGT excretion between CKD etiologies can probably be explained by differences in filtration and/or tubular reabsorption (Kobori et al., 2008; Urushihara et al., 2010; Nishiyama et al., 2011; Park et al., 2015b; Salih et al., 2017; Tang et al., 2018; Kim et al., 2019; Ohashi et al., 2020).

**TABLE 3:** Urinary angiotensinogen in patients with kidney disease

Chronic Kidney Disease
UAGT correlates with ACR (Yamamoto et al., 2007; Kobori et al., 2008; Kim et al., 2011a; Lee et al., 2011; Mills et al., 2012; Zhang et al., 2013; Jeon et al., 2020). Lower eGFR is associated with higher UAGT (Yamamoto et al., 2007; Kobori et al., 2008; Kim et al., 2011a; Mills et al., 2012; Zhang et al., 2013; Jeon et al., 2020). UAGT correlates with intrarenal Ang II or AGT (Yamamoto et al., 2007; Zhang et al., 2013). UAGT correlates with severity of kidney damage on biopsy (Kim et al., 2011a). UAGT is increased compared with healthy controls (Kobori et al., 2008; Mills et al., 2012; Zhang et al., 2013). UAGT predicts effect of (Jeon et al., 2020) and is reduced after ACEI or ARB (Lee et al., 2011).
Diabetic Kidney Disease
UAGT correlates with ACR (Terami et al., 2013; Satirapoj et al., 2014; Park et al., 2015b; Zhuang et al., 2015; Lee et al., 2017; Ba Aqeel et al., 2019). Lower eGFR is associated with higher UAGT (Terami et al., 2013; Satirapoj et al., 2014; Zhuang et al., 2015). Lower eGFR is not associated with higher UAGT (Park et al., 2015b). UAGT correlates with tubular damage markers (Terami et al., 2013). UAGT is higher in DKD compared with controls (Satirapoj et al., 2014) or type 1 diabetes mellitus without DKD (Wysocki et al., 2017a). UAGT is also increased in DKD patients without overt albuminuria (Satirapoj et al., 2014; Zhuang et al., 2015). UAGT is higher in DKD compared with CKD (Park et al., 2015b). UAGT does not predict disease progression after correction for ACR (Lee et al., 2017; Ba Aqeel et al., 2019). UAGT predicts disease progression after correction for ACR and other clinical parameters (Satirapoj et al., 2019).
IgA Nephropathy
UAGT correlated with ACR (Urushihara et al., 2010; Kim et al., 2011b; Konishi et al., 2011; Nishiyama et al., 2011; Jang et al., 2012; Urushihara et al., 2015). Lower eGFR is associated with higher UAGT (Kim et al., 2011b; Jang et al., 2012). No association between eGFR and UAGT (Ohashi et al., 2020). UAGT is higher compared with healthy controls (Urushihara et al., 2010; Kim et al., 2011b; Nishiyama et al., 2011) and other causes of CKD (Kim et al., 2011b). UAGT correlates to disease severity (Konishi et al., 2011; Jang et al., 2012; Urushihara et al., 2015) and Ang II/AGT (Nishiyama et al., 2011; Jang et al., 2012) in kidney biopsies. UAGT decreases after RAS inhibition (Urushihara et al., 2010, 2015; Nishiyama et al., 2011). UAGT predicts eGFR and proteinuria, no correction for ACR (Jang et al., 2012).
Autosomal Dominant Polycystic Kidney Disease
UAGT correlated with ACR (Kocyigit et al., 2013; Kurultak et al., 2014; Salih et al., 2017; Kim et al., 2019; Park et al., 2020). Lower eGFR is associated with higher UAGT (Park et al., 2015a). No association between eGFR and UAGT (Kocyigit et al., 2013; Salih et al., 2017; Park et al., 2020). Positive (Park et al., 2015a) or no correlation (Salih et al., 2017; Park et al., 2020) of UAGT with total kidney volume. UAGT increased compared with healthy controls (Kocyigit et al., 2013; Kurultak et al., 2014) and CKD (Salih et al., 2017; Kim et al., 2019). UAGT predicts disease progression (Park et al., 2020). UAGT correlated to serum potassium (Kim et al., 2019).
Miscellaneous
In patients with nephrotic range proteinuria, UAGT correlates with proteinuria (Jang et al., 2014; Tang et al., 2018). Plasma AGT correlates with UAGT in patients with nephrotic syndrome (Jang et al., 2014). No correlation between ACR and UAGT in minimal change disease (Tang et al., 2018). UAGT is increased in patients with AA amyloidosis compared with healthy controls (Kutlugün et al., 2012). The AGT/ACR ratio differentiates between TIN and IgA nephropathy (Ohashi et al., 2020).

ACR, albumin/creatinine ratio; TIN, tubulointerstitial nephritis; UAGT, urinary angiotensinogen.

Urinary AGT is widely used as a marker for intrarenal Ang II generation and therefore intrarenal RAS activity. In almost all patient cohorts with CKD in which urinary AGT excretion was measured, it was closely correlated to urinary albumin excretion (Table 3). Albuminuria is an established predictor for CKD progression and a treatment target, yet whether urinary AGT excretion has added value compared with albuminuria is uncertain. In two studies in patients with DKD (Lee et al., 2017; Ba Aqeel et al., 2019), the predictive effect of urinary AGT excretion on estimated GFR (eGFR) decline was lost after correction for albuminuria. In two cohorts of patients with ADPKD (Kim et al., 2019; Park et al., 2020), AGT excretion was also a predictor of disease progression. However, one study did not include total kidney volume and albuminuria in their model (Kim et al., 2019), and the other study did not correct for known ADPKD risk factors at all (Park et al., 2020). In patients with AKI, AGT and Ang II immunoreactivity in biopsies and urinary AGT excretion were increased and related to AKI severity (Cao et al., 2016). Furthermore, baseline urinary AGT excretion predicted the likelihood of AKI after cardiac surgery (Alge et al., 2013a), in decompensated heart failure (Yang et al., 2015), and in patients admitted to the ICU (Alge et al., 2013b). However, also in these studies, urinary albumin excretion was not included in the models. Given that AGT, like albumin and renin, is filtered by the glomerulus and that all three proteins are subsequently reabsorbed via megalin, with AGT and renin subsequently potentially contributing to renal Ang II generation, it is not surprising that urinary AGT is a proxy for renal RAS activity and future kidney injury. Yet, the same is probably true for urinary albumin. Given that the proximal tubule generates minute amounts of AGT, which are released directly into urine without being converted to Ang II, one possibility is that at a very early stage of CKD/DKD, when filtration/reabsorption are still normal, this AGT already reflects kidney damage prior to any rise in urinary albumin (Saito et al., 2009). Future studies should carefully evaluate this possibility, quantifying urinary and circulating AGT and albumin simultaneously. At later CKD stages, this possibility becomes unlikely since then the contribution of filtered AGT will become overwhelming.

### C. Metabolic Disorders

Several studies propose a role for the intrarenal RAS in obesity and insulin resistance. Compared with lean control rats, obese Zucker rats demonstrated decreased renin expression and increased AT_2_ receptor expression in the kidneys, whereas ACE, AT_1_ receptor, and Mas receptor expression were unchanged (Ali et al., 2013). Although there were no changes in kidney Ang I and II levels, ACE2 expression and activity was reduced in obese rats. Further, treatment with an AT_2_ receptor agonist increased renal ACE2 expression and activity, Ang-(1-7) levels, and Mas receptor expression, leading to natriuresis and lower blood pressure (Ali et al., 2013). Conversely, AT_1_ receptor is increased in obese Zucker rats, whereas treatment with ARBs attenuated the renal expression of inflammatory and fibrosis markers and kidney injury (Xu et al., 2005).

Mice with metabolic syndrome characterized by insulin resistance, visceral obesity, and postprandial hyperglycemia due to a null mutation in carcinoembryonic antigen-related cell adhesion molecule 1 display enhanced expression of RAS components at the level of the renal tubule, resulting in increased renal interstitial fluid Ang II levels (Huang et al., 2013). These mice were also hypertensive and had albuminuria. Notably, all of these abnormalities were exacerbated by high fat intake (Li et al., 2015). Similarly, isolated perfused kidneys from rats treated with a high fructose diet (a model of metabolic syndrome) demonstrate a 2- to 3-fold increase in kidney Ang I and II content without significant differences in Ang-(1-7), ACE, and neprilysin (Yokota et al., 2018). Collectively, these studies suggest that the intrarenal RAS may be activated and could contribute to kidney injury in obesity and insulin resistance.

Consistent with the above notion, multiple clinical studies using ACEIs and ARBs have demonstrated beneficial effects with regard to insulin sensitivity and the development of new-onset diabetes in patients with obesity/metabolic syndrome (Vermes et al., 2003; Tocci et al., 2011). However, it still remains to be determined whether this involves interference with RAS components at the level of the human kidney.

### D. In Vitro Models for Human Disease: Kidney Organoids

**Fig. 8:**
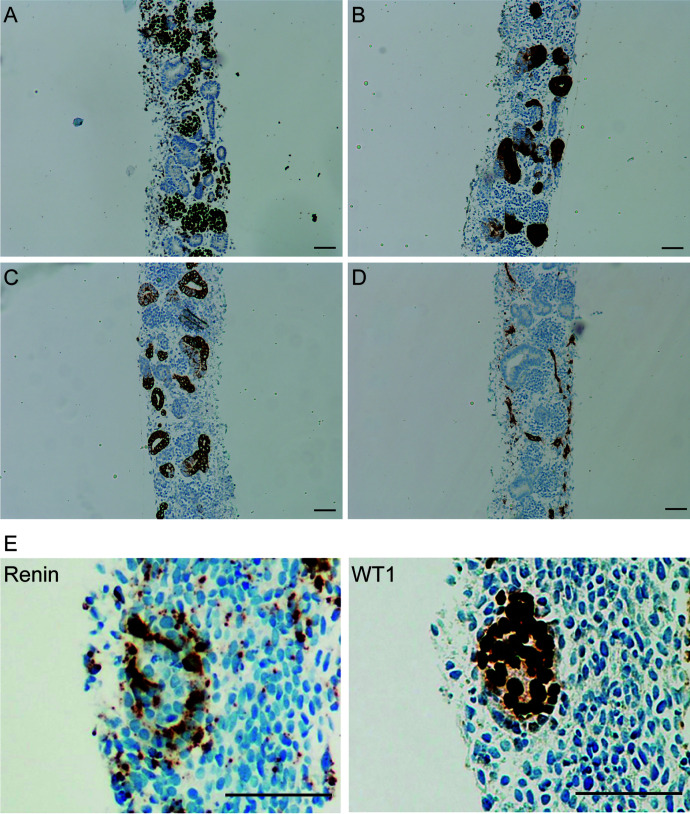
Immunohistochemical characterization of human induced pluripotent stem cell-derived kidney organoids. (A) Wilms’ tumor suppressor gene 1 (WT1) staining of glomerular structures; (B) Villin1 staining of proximal tubular structures; (C) E-cadherin staining of distal tubular structures; (D) CD31 staining of endothelial cells; (E) Renin staining, localized around WT1+ area. Organoids were generated as described by Shankar et al. (2021). Scale bar = 50 *μ*m.

In vitro models are valuable tools for studying human kidney disease development, progression, and treatment. The increased knowledge on human embryological kidney development and the advance of cell culture technologies have boosted the generation of pluripotent stem cell–derived kidney organoids. These organoids are formed by timely stimulation of Wnt signaling, addition of fibrobast growth factor 9, and 3D-culturing conditions of embryonic stem cells or induced pluripotent stem cells and exhibit remarkable resemblance with first trimester fetal kidney (Morizane et al., 2015; Takasato et al., 2015). Modifications to the extracellular environment can further advance the differentiation status of kidney organoids and drive them into a state that transcriptionally resembles second trimester kidney (Garreta et al., 2019). Morphologically, kidney organoids display glomerular, proximal tubular, and distal tubular structures and contain endothelial cells (Fig. 8).

Kidney organoids can be generated from induced pluripotent stem cells derived from kidney disease patients, thereby forming a model to study the impact of genetic disease on kidney development (Forbes et al., 2018). In addition, kidney disease can be modeled in kidney organoids through CRISPR/Cas9 technology. This has been demonstrated by knocking out the polycystic kidney disease genes *PKD1* or *PKD2,* which led to the formation of cysts in organoids (Freedman et al., 2015). The development of automated multiwell kidney organoid culture technology allows for high-throughput toxicity and efficacy screening of novel drugs for treatment of kidney disease (Czerniecki et al., 2018).

Kidney organoids contain a relatively large stromal cell compartment. We recently demonstrated that subsets of stromal cells in kidney organoids have the capacity to produce and secrete enzymatically active renin (Shankar et al., 2021). Renin production could be drastically stimulated by intracellular cAMP elevation and was maintained for at least 2 months after implantation of kidney organoids in a mouse model. Several other components of the RAS are also expressed in kidney organoids, including AGT, AT_1_ receptor, AT_2_ receptor, neprilysin, and ACE. Kidney organoids also express ACE2, which sparked research into the role of renal ACE2 in COVID-19. It was demonstrated that SARS-CoV-2 virus can infect kidney organoids and that human recombinant soluble ACE2 can inhibit infection (Monteil et al., 2020; Wysocki et al., 2021). Kidney organoids thus possess properties that allow studies to the RAS in a human in vitro setting, from exploring RAS during kidney development to the impact of disease-specific pluripotent stem cells on RAS and pharmacological intervention.

Although pluripotent stem cells have proven their use for studying in vitro nephrogenesis, adult stem cells derived from human kidney or urine have been demonstrated to be capable of forming kidney tubuloids in a dish. Such tubuloids represent proximal and distal tubular structures and can be propagated in culture for multiple months (Schutgens et al., 2019). As these structures are in a more matured stage of development, they are especially useful for studying kidney repair processes. Tubuloids are likely to express RAS components found in the adult kidney and therefore represent a yet unexplored model for studying pharmacological interventions in the RAS in a human in vitro setting.

## V. Renoprotective Drugs Affecting Kidney Angiotensin Levels

### A. RAS Inhibitors

**Fig. 9:**
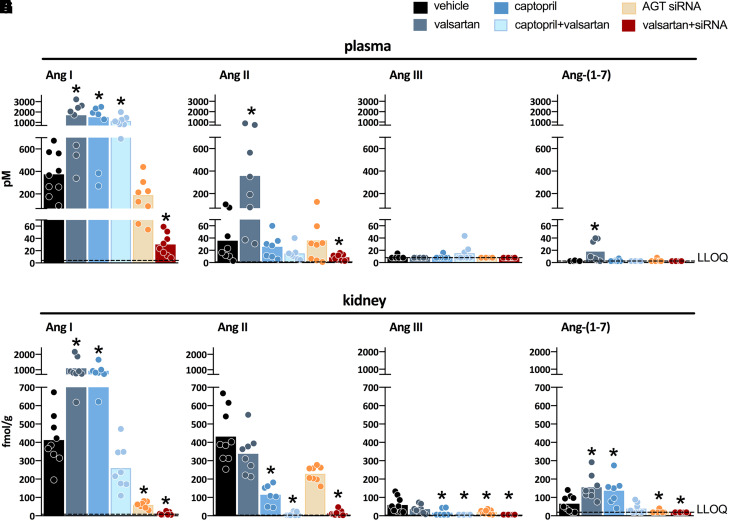
Effect of angiotensin (Ang) II type 1 receptor blockade with valsartan, angiotensin-converting enzyme inhibition with captopril, angiotensinogen (AGT) siRNA, or their combination during 4 weeks on the levels of Ang I, Ang II, Ang III, and Ang-(1-7) in blood and kidney of spontaneously hypertensive rats. Modified from Uijl et al. (2019). *P < 0.05 vs. vehicle. LLOQ, lower limit of quantification.

**Fig. 10:**
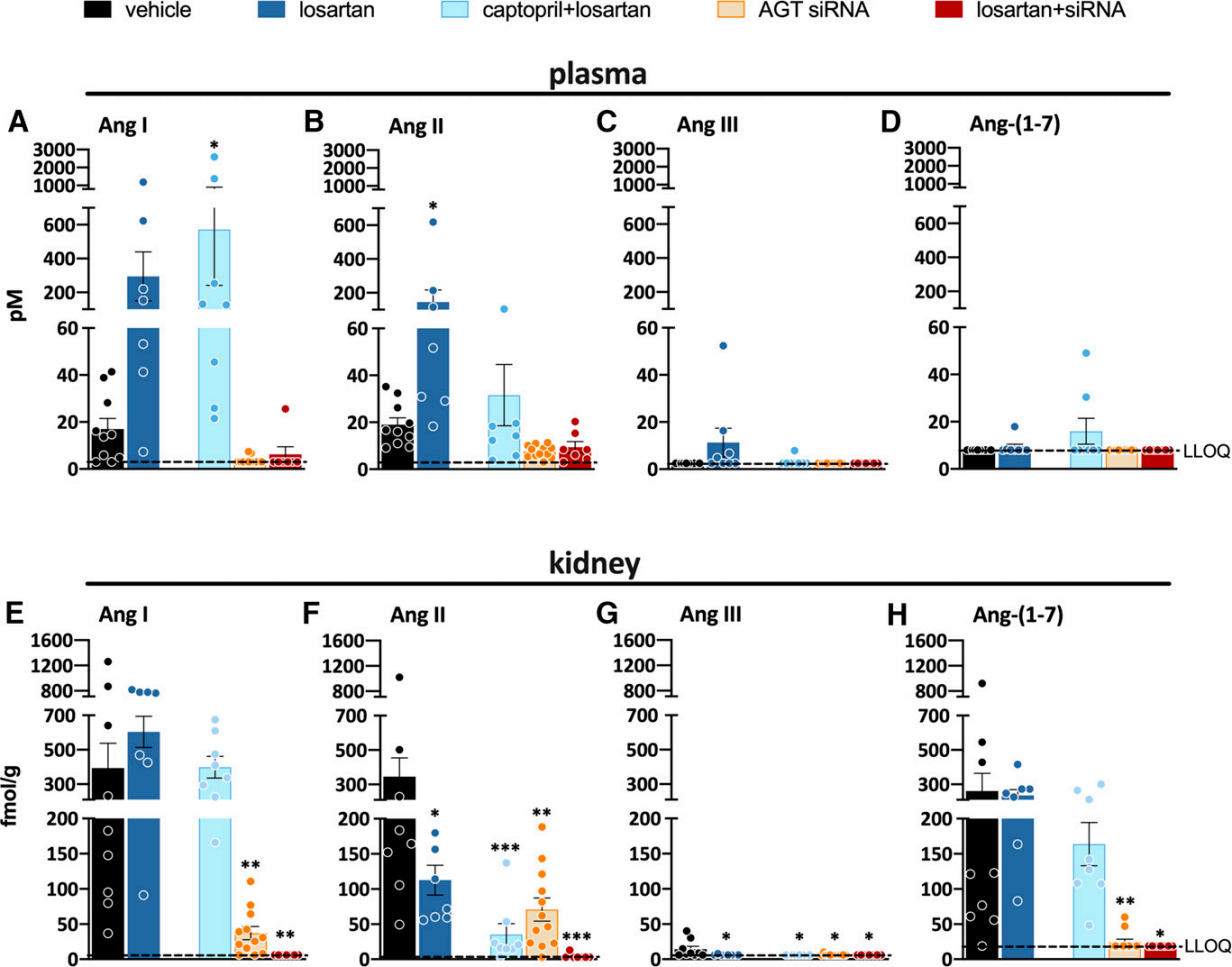
Effect of angiotensin (Ang) II type 1 receptor blockade with losartan, angiotensin-converting enzyme inhibition with captopril, angiotensinogen (AGT) siRNA, or their combination during 4 weeks on the levels of Ang I, Ang II, Ang III, and Ang-(1-7) in blood and kidney of five-sixths nephrectomy rats. Modified from Bovée et al. (2021). *P < 0.05, **P < 0.01, ***P < 0.001 vs. vehicle. LLOQ, lower limit of quantification.

Blocking the RAS is currently possible at four levels: renin (renin inhibitors), ACE (ACEIs), AT_1_ receptors (ARBs), and AGT (siRNA or antisense oligonucleotides), although the latter approach is not yet available in the clinic. The beneficial effects of the ‘classic’ RAS blockers (ACEIs and ARBs) are widely accepted. Figures 9 and 10 show the changes in the four main angiotensin metabolites, determined by liquid chromatography-tandem mass-spectrometry, after ACEIs, ARBs, AGT siRNA, or their combination in both SHRs and five-sixths nephrectomy rats (Uijl et al., 2019; Bovée et al., 2021). The latter rats display both hypertension and kidney dysfunction. Results in the two models were highly similar.

**Fig. 11:**
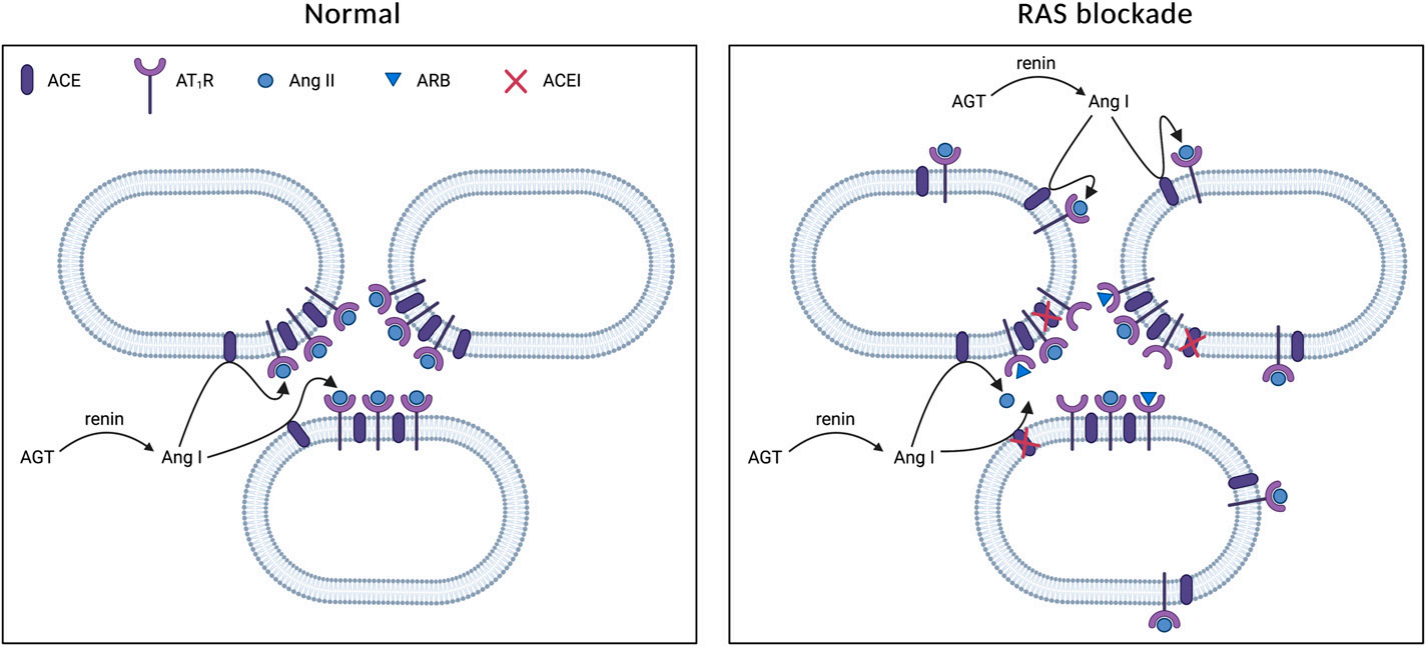
Proposed mechanism of how renin-angiotensin system (RAS) blockers work, even when not lowering the tissue levels of angiotensin (Ang) II. Normally, Ang II generation occurs in a highly localized manner by angiotensin-converting enzyme (ACE), allowing local Ang II type 1 receptor (AT_1_R) stimulation. After treatment with an AT_1_R (ARB) or ACE inhibitor (ACEI), this is no longer possible, although upregulation of ACE and AT_1_R expression at alternative sites may occur, thus still allowing Ang II formation and AT_1_R stimulation but in a less efficient (‘diluted’) manner.

ACEIs lower renal Ang II but not plasma Ang II, both alone and in combination with an ARB. ACEIs increase Ang I in both kidney and blood, and apparently this is sufficient to restore circulating Ang II but not renal Ang II. This indicates that the kidney is very sensitive to ACE inhibition, as has been suggested before, also in comparison with other organs (Campbell et al., 1995b; Campbell, 1996). The observation that the Ang II/I ratio (the best in vivo indicator of ACE activity) dropped by >90% after ACEIs argues against a significant contribution of other converting enzymes in the kidney. Here we need to consider that ACE inhibition in vivo is unlikely to reach 100%, and thus the remaining <10% renal Ang I-II converting activity likely reflects the small fraction of ACE that is not inhibited (i.e., it does not by definition support a role for other converting enzymes like chymase). These data also indicate that modest ACE upregulation, even if occurring, would have marginal consequences. This is not surprising since the blockade of >90% will also apply to any de novo synthesized ACE. To truly overcome such blockade, 10- to 20-fold rises in ACE rises are required. In reality, ACE upregulation is usually in the order of 2-fold or less (Gonzalez-Villalobos et al., 2013). The only RAS component capable of matching a >90% blockade is renin, allowing immediate and substantial upregulation of Ang I and therefore ongoing Ang II generation even when only <10% of ACE activity is left. A limit in its capacity to rise has not yet been described, and rises of several 100-fold are easily feasible (Balcarek et al., 2014). This is related to the fact that VSMCs along the renal arterioles can transform into renin cells (see section II.A). Hence, depending on the ACEI dose and the degree of renin upregulation, which may differ per condition (e.g., renin rises are usually more substantial during a low-salt diet), it is possible that occasionally renal Ang II levels do return to normal after long-term ACEI treatment. If so, the question arises as to why ACE inhibition would be effective at all. Here one might argue that Ang II generation normally occurs in a highly localized manner and is followed by its rapid binding to surrounding AT receptors to exert effects. After ACEI treatment, rapidly reaching high local Ang II levels is no longer possible. Higher renin levels, combined with modest ACE upregulation at additional sites, may help to restore the total amount of Ang II that is generated. However, the generation now occurs more evenly spread and no longer regionally, and thus AT_1_ receptor stimulation occurs less efficiently (Fig. 11).

Data on the effect of renin inhibition on renal tissue Ang I and II levels are scarce. This is related to the fact that the only renin inhibitor that is currently clinically available, aliskiren, is a human renin inhibitor with limited efficacy for rodent renin. Moreover, its oral bioavailability is very low (Danser, 2007). Unfortunately, renin, unlike ACE and the AT_1_ receptor, is highly species specific, and thus ACEIs and ARBs can be easily tested in rodents whereas renin inhibitors cannot, except of course in rodents that are transgenic for human renin and AGT (Pilz et al., 2005). Theoretically, one would expect a renin inhibitor to lower Ang II (and Ang I) even better since such an inhibitor would also prevent the consequences of the rises in renin that will occur during RAS blockade. Although aliskiren did indeed lower Ang I and II acutely in the SHR and Sprague Dawley kidney (van Esch et al., 2010a; Campbell et al., 2011), the fact that only very high doses were tested, whereas no information was obtained on the changes in the other angiotensin metabolites, precludes clear conclusions on its efficacy with regard to renal angiotensin levels. The limited evidence that is available supports that aliskiren is at least as renoprotective as other RAS blockers, both in animals and in humans (Pilz et al., 2005; Fisher et al., 2008; van Esch et al., 2010a; Campbell et al., 2011).

Figures 9 and 10 show that ARBs increase circulating Ang I and II to the same degree, while not affecting renal Ang II, and at most increase renal Ang I. Hence, the renal Ang II/I ratio usually decreases substantially after ARB treatment. These data reflect the well known rise in RAS activity that will occur after ARB treatment. Yet, although there is much more Ang II, it cannot bind to its receptors at renal tissue sites due to the fact that the receptors are now largely occupied by the ARB. Thus, a high degree of local AT_1_ receptor stimulation is no longer achievable, and at most the total tissue Ang II levels remain the same, but this Ang II is now more evenly spread (Fig. 11). Ang II may also bind to its AT_2_ receptor and exert anti-AT_1_ receptor effects (see section III.B).

In blood, both Ang-(1-7) and Ang III remained low or undetectable during ACEI and ARB treatment (Figs. 9 and 10). The same is true for Ang III in the kidney, the level of which, if anything, decreased even further during treatment. This raises doubt about the concept that Ang III is the endogenous agonist of the AT_2_ receptor (Padia et al., 2008; van Esch et al., 2008). In SHRs, renal Ang-(1-7) levels increase during ACEI and ARB treatment, in parallel with Ang I, but this was not seen in the five-sixths nephrectomy rat, where Ang-(1-7) levels remained unchanged. Nevertheless, this implies that during both types of treatment the Ang-(1-7)/Ang II ratio generally increased, favoring effects of the former (see section III.C).

As discussed in section II.C, the use of AGT siRNA has demonstrated that renal angiotensin generation depends on hepatic AGT. Remarkably, despite lowering plasma AGT by >95%, siRNA did not alter blood Ang I or II in SHRs, whereas after siRNA only a modest drop in circulating Ang II was seen in the five-sixths nephrectomy model. Yet, at the level of the kidney, all angiotensin metabolites went down after siRNA in both models, and in combination with an ARB they quite often almost entirely disappeared (Figs. 9 and 10). The drop in renal tissue Ang II directly translated into a reduction in proteinuria in the five-sixths nephrectomy rat (Bovée et al., 2021). In blood, dual RAS blockade also lowered Ang II but not as completely as in the kidney. These data therefore illustrate the great sensitivity of the kidney to AGT suppression. This is probably related to the fact that the renal AGT supply relies entirely on a complicated AGT uptake process, depending on (normally) very high circulating AGT levels in the Michael-Menten constant (K_m_) range. Dropping these by >95% hampers sufficient uptake and will affect the possibility of generating sufficient angiotensins at tissue sites. A similar situation has been observed in subjects with end-stage heart failure receiving left ventricular assist device support (Klotz et al., 2009). Their renin levels were so high (immediately cleaving the small quantities of AGT that were still left) that cardiac AGT became depleted, thus no longer allowing Ang II generation at cardiac tissue sites. The fact that a >95% drop in AGT still allowed circulating Ang II levels to be in the normal range is yet another example of the versatility of the RAS to restore its activity, at least in blood. This relied on a very substantial rise in renin and thus made the rat circulating RAS look temporarily like the mouse circulating RAS (which is characterized by AGT levels in the order of a few percent of those in humans and rats).

Finally, although it was initially believed that the more RAS blockade the better in patients who require RAS blocker treatment, this turned out not to be true (Parving et al., 2012; Fried et al., 2013). Too much RAS blockade resulted in hyperkalemia, hypotension, and renal failure (i.e., the predictable consequences of RAS annihilation), especially when renal function was already compromised when the additional blocker was added. The beneficial effects did not increase concomitantly. This outcome is due to the fact that kidney function (particularly glomerular filtration) depends on the RAS (Balcarek et al., 2014). Hence, a patient requires optimal rather than maximal RAS blockade (Danser and van den Meiracker, 2015). This does not imply that the use of more than one RAS blocker should now always be avoided: it may still be needed to obtain sufficient RAS suppression in an individual patient.

A condition in which RAS blockers should not be used is pregnancy. This is because the RAS is needed for the development of the fetal kidney since Ang II acts as an important growth factor (e.g., for proximal tubular cells) (Tufro-McReddie et al., 1994). Indeed, AGT KO mice display renal morphologic abnormalities such as tubulointerstitial lesions and papillary atrophy, resulting in an impaired urine-concentrating ability and low blood pressure (Niimura et al., 1995; Kihara et al., 1998). Similarly, mutations in RAS genes (renin, AGT, ACE, and AT_1_ receptor) resulting in the absence or inefficacy of Ang II associated with autosomal recessive renal tubular dysgenesis, hypotension, and anuria (Gribouval et al., 2005; Richer et al., 2015).

### B. Soluble ACE2

An alternative approach to deal with excessive renal Ang II levels is to increase its degradation by amplifying ACE2 activity (Wysocki et al., 2019; Marquez et al., 2021). This is particularly relevant given the decreased ACE2 activity in several kidney pathologies, as discussed in section II.E. Different approaches have been used to amplify ACE2 activity to achieve a therapeutic benefit or as proof-of-concept studies. This includes viral delivery systems (lenti-, adeno- and adeno-associated virus), minicircle DNA delivery, and the administration of recombinant proteins (Marquez et al., 2021). Here, we briefly mention the therapeutic potential of recombinant ACE2 proteins, focusing on kidney disease and COVID-19.

**Fig. 12:**
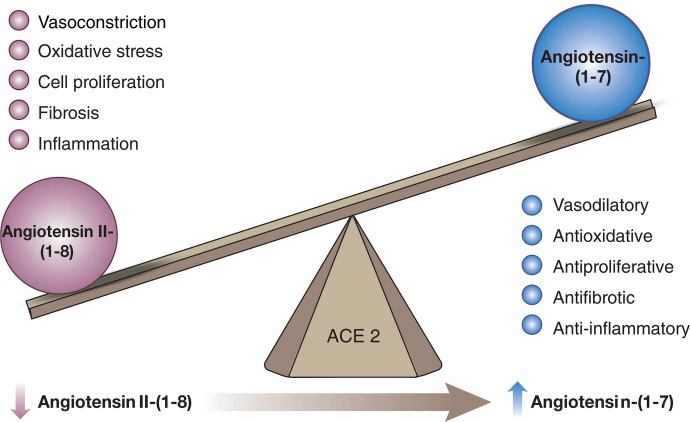
Scheme of the biologic actions of angiotensin (Ang) II and Ang-(1-7) and the role of ACE2.

The therapeutic benefit of ACE2 amplification presumably depends on both the dissipation of excess Ang II and the formation of Ang-(1-7) (Fig. 12). Wysocki et al. (2010) demonstrated the effectiveness of both human and mouse recombinant soluble ACE2 in Ang II–dependent hypertension (Ye et al., 2012). To accomplish this potential therapeutic action, both the native recombinant soluble ACE2 protein (740 amino acids) and the full-length ACE2 gene have been used. In STZ-induced diabetes, overexpression of human ACE2 in podocytes increased podocyte numbers and delayed the onset of albuminuria (Nadarajah et al., 2012). In a rat model of STZ-induced diabetes, ACE2 expression by adenovirus reduced systolic blood pressure, urinary albumin excretion, and creatinine clearance and improved the glomerulosclerosis index (Liu et al., 2011a). In STZ-treated diabetic mice, both minicircle DNA gene delivery of soluble ACE2 and intraperitoneal injection of recombinant mouse soluble ACE2 protein increased plasma ACE2 levels; however, there was no effect on the hyperfiltration typical of early diabetes or glomerular lesions (Wysocki et al., 2017b). The large molecular size of the native soluble protein was considered the limiting factor when targeting the kidney directly since kidney and urinary ACE2 levels were not increased after injection of recombinant ACE2/minicircle DNA delivery of soluble ACE2 in these mice (Wysocki et al., 2013, 2017b). Conversely, in Col4a3 KO mice, a model of experimentally induced Alport syndrome, urinary ACE2 activity increased markedly after injection of recombinant mouse ACE2 (Wysocki et al., 2017b). Moreover, this injection improved kidney fibrosis (Bae et al., 2017). Systemically administered native recombinant ACE2 normally cannot cross the glomerular filtration barrier due to its large molecular size (120–130 kDa). Yet, in this model of Alport syndrome it does pass through, owing to the markedly disrupted glomerular filtration barrier (Wysocki et al., 2017b). To overcome this limitation for treatment of early kidney disease, novel recombinant soluble mouse ACE2 proteins of smaller molecular size (605 to 619 amino acids) were developed (Wysocki et al., 2019). These shorter truncates are systemically active and amenable to glomerular filtration as shown by increased kidney ACE2 activity in ACE2 KO mice after ACE2 1-619 infusion, whereas there was no increase in animals infused with native ACE2 1-740 (Wysocki et al., 2019). To increase the half-life of the shorter ACE2 truncates, Wysocki et al. (2021) fused a human counterpart of ACE2 with an albumin binding domain. The resulting fusion protein has a markedly extended half-life and is now being tested in preclinical models of kidney disease and COVID-19.

Given that ACE2 is the main cell entry receptor for SARS-CoV-2, there may be a role for soluble ACE2 proteins to act as a decoy in COVID-19 (Batlle et al., 2020b; Wysocki et al., 2021; Hassler et al., 2022), preventing viral internalization. A consequence of SARS-CoV-2 infection is internalization of ACE2, which causes a decrease in membrane-bound ACE2 (Kuba et al., 2005). This should result in loss of enzymatic ACE2 activity, which would favor the accumulation of proinflammatory peptides like Ang II, thereby increasing the risk of local injury. Indeed, in ACE2 KO mice, acid aspiration–induced lung injury was more severe than in wild-type mice (Imai et al., 2005). The enzymatic action of administered soluble ACE2 proteins, in addition to the decoy effect, might help to prevent accumulation of these harmful peptides (Monteil et al., 2020; Wysocki et al., 2021; Hassler et al., 2022).

The decoy effect of several soluble ACE2 proteins has now been tested in kidney organoids and animal models of SARS-CoV-2 infection (Monteil et al., 2020; Wysocki et al., 2021; Hassler et al., 2022). A soluble ACE2 variant with increased binding affinity for SARS-CoV-2 infection prevented severe disease, lowered viral lung and brain titers and markedly improved lung histology in k18hACE2 mice, a model of otherwise lethal SARS-CoV-2 infection (Hassler et al., 2022). The kidneys in this model of SARS-CoV-2 infection, however, are not well studied, but AKI is a complication frequently seen in patients with severe COVID-19 (Batlle et al., 2020a). In the aforementioned study, some k18hACE2 mice 6 days post–SARS-CoV-2 infection did develop severe proximal tubular injury, based on histologic findings, whereas soluble ACE2 markedly improved kidney histology (Hassler et al., 2022). The only clinical trial based on the systemic administration of a native soluble ACE2 protein approved for clinical use in COVID-19 patients did not meet the primary endpoints, a composite of all-cause death or invasive mechanical ventilation (NCT04335136). One possibility for these results is that the systemic route is likely not as effective as the intranasal route to neutralize SARS-CoV-2, and in addition the short duration of action of this protein may not afford 24-hour protection to intercept SARS-CoV-2 from getting into cells.

### C. Neprilysin Inhibitors: ARNI

Dual ARNI is currently registered for the treatment of patients with heart failure (Ponikowski et al., 2016), in whom studies showed a substantial reduction in mortality and hospitalization (McMurray et al., 2014). Sacubitril/valsartan (LCZ696) is the first-in-class ARNI. In vivo, sacubitril is rapidly metabolized into the active neutral endopeptidase/neprilysin (NEP) inhibitor sacubitrilat. NEP degrades both vasodilators (e.g., natriuretic peptides, bradykinin, adrenomedullin) and vasoconstrictors (e.g., Ang II, endothelin-1). Therefore, the effect of single NEP inhibition is unpredictable, as it depends on the dominance of either vasodilators or vasoconstrictors (Ando et al., 1995). Dual therapy, however, guarantees the beneficial effects of increasing natriuretic peptides (e.g., increased diuresis, natriuresis, and vasodilation) because simultaneous blockade of the AT_1_ receptor with valsartan counteracts the NEP inhibitor–induced rise in Ang II.

Recent studies have investigated whether sacubitril/valsartan might also be renoprotective. When compared with conventional RAS inhibitors, sacubitril/valsartan slowed the rate at which kidney function declined in patients with heart failure, both in those with and without concurrent CKD (Voors et al., 2015; Damman et al., 2018; Mc Causland et al., 2020). Unexpectedly, sacubitril/valsartan-treated patients displayed a slight increase in urinary albumin-creatinine ratio (Voors et al., 2015; Damman et al., 2018). However, baseline albuminuria was very low in these studies, and sacubitril/valsartan may reduce proteinuria particularly in patients with macroalbuminuria (Ito et al., 2015). In contrast, in the United Kingdom Heart and Renal Protection-III trial, sacubitril/valsartan had similar effects to irbesartan on both measured GFR and albuminuria in patients with moderate-to-severe CKD, even though blood pressure control and neurohumoral markers of cardiac dysfunction were improved superiorly (Haynes et al., 2018). CKD was caused by diabetes in only a subset of the participants, whereas sacubitril/valsartan appears to be most effective at protecting kidney function in this population (Packer et al., 2018). Indeed, in rats with severe diabetic, hypertensive kidney damage, ARNI induced a greater reduction in proteinuria and glomerulosclerosis than ARB (Roksnoer et al., 2016c). This appeared to be due to preserved podocyte integrity and was independent of blood pressure changes (Uijl et al., 2020). It involved the upregulation of atrial natriuretic peptide, which increased nephrin expression and suppressed both transient receptor potential canonical 6 and regulator of calcineurin 1. Superior renoprotective effects of sacubitril/valsartan versus valsartan were also seen in obese Zucker rats and the five-sixths nephrectomy model (Jing et al., 2017; Habibi et al., 2019).

A critical question remains as to whether the ARNI effects are simply the added consequence of blocking AT_1_ receptors and increasing natriuretic peptide levels or if they involve an additional (synergistic?) interaction between the two systems (e.g., in the kidney). Natriuretic peptides directly suppress renin release from JG cells (Henrich et al., 1988). Furthermore, if NEP inhibition truly increases Ang II levels, this should also suppress renin. Currently, knowledge on this topic after ARNI treatment is entirely lacking. In a study in transgenic (mRen2)27 rats (i.e., hypertensive rats that overexpress the mouse *Ren2* gene), NEP inhibition did not affect renin (Roksnoer et al., 2015). This could be due to the fact that in these rats, renin not only originates in the kidney. In human kidney homogenates, NEP has been suggested to generate Ang-(1-7) from Ang I (Kaltenecker et al., 2020). Remarkably, NEP levels were diminished in CKD kidneys compared with healthy kidney donors, implying that Ang-(1-7) would be low in CKD. Assuming that Ang-(1-7) exerts a beneficial effect, this finding would argue against the use of NEP inhibition in such patients, in full contrast with the abovementioned clinical trials. Here it is important to stress that data obtained in homogenates are unlikely to represent in vivo physiology. Indeed, treatment of rats with a NEP inhibitor did not alter renal Ang-(1-7) levels, neither at baseline nor on top of ARB treatment (Roksnoer et al., 2015). Thus, in reality, the in vivo contribution of NEP to Ang I-Ang-(1-7) conversion may be negligible. Taken together, the evidence obtained until now does not favor a synergistic interaction between AT_1_ receptor antagonism and NEP inhibition at the level of the kidney.

### D. Cyclooxygenase Inhibitors

**Fig. 13:**
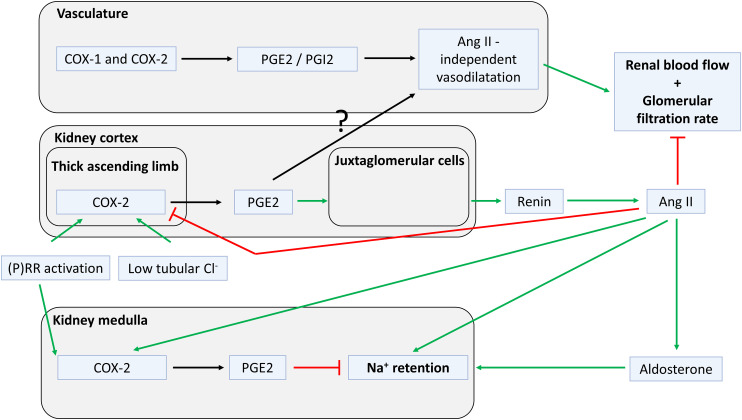
Interactions between prostaglandins and the renin-angiotensin system (RAS) in the kidney. Firstly, in the vasculature, prostaglandin E2 (PGE2) and I2 (PGI2) cause vasodilatation, counteracting the vasoconstriction of angiotensin (Ang) II. Secondly, in the kidney cortex, PGE2 is produced when hypovolemia occurs and subsequently induces renin production by juxtaglomerular cells. Finally, in the kidney medulla, PGE2 counteracts the effects of Ang II on sodium retention.

Prostaglandins are produced from arachidonic acid by COX-1 and -2 in combination with terminal prostaglandin synthases. In the kidney and its vasculature, both COX-1 and COX-2 are present. COX-2 activity is further inducible by changes in salt intake and RAS activity with a differential regulation between cortex and medulla (Fig. 13) (Yang et al., 1998). PGE2 is the most abundantly produced prostaglandin in the kidney. PGE2 plays an important role in the regulation of renin release, kidney blood flow, tubular water and salt handling, kidney development, and the proliferative response to injury. COX-1 and COX-2 are inhibited by nonsteroidal anti-inflammatory drugs (NSAIDs). Selective inhibitors of COX-2 were developed to avoid the gastrointestinal side effects of NSAIDs. Both NSAIDs and COX-2 inhibitors are associated with increased cardiovascular mortality, hypertension, and nephrotoxicity, especially in hypovolemic subjects (Bhala et al., 2013). Interestingly, COX inhibition can also be renoprotective in hyperfiltrating and albuminuric CKD patients by reducing glomerular perfusion pressure, with similar short-term benefits as RAS inhibition (Vogt et al., 2009). To further mitigate the systemic side effects of COX inhibition, specific PGE2 (EP) receptor agonists and antagonists are currently being developed for different indications and used in animal models of kidney disease and hypertension (Markovič et al., 2017). In the kidney, the effects of the COX-PGE2 axis and the RAS are closely linked in regulating renovascular resistance, renin secretion, and sodium excretion (Nasrallah et al., 2014).

In the glomerulus, COX-1–derived PGI2 and PGE2 cause vasodilation of the afferent arteriole and maintain kidney blood flow and GFR in situations with active vasoconstrictors through activation of the EP4 receptor (Tang et al., 2000). Even though the vasodilatory effect of PGE2 depends on concurrent vasoconstriction by other mediators, the effect of COX inhibition on renovascular flow is independent of intrarenal Ang II. This was shown in a study that compared two groups of rats with similar cortical COX activity but different Ang II levels (Green et al., 2010). Rats on a low-sodium diet had increased cortical COX and kidney Ang II levels, whereas rats treated with ACEIs had increased cortical COX activity but low kidney Ang II levels. After COX inhibition, eGFR and kidney blood flow were reduced similarly in both groups, providing evidence for the independence of PGI2 and PGE2 effects on vascular tone from prevailing RAS activity (Green et al., 2010).

The COX-PGE2 axis and its interaction with the RAS are also essential for normal kidney development. COX-2 KO mice and neonatal mice treated with COX-2 inhibitors develop kidney failure characterized by cortical damage, with immature glomeruli and tubules (Dinchuk et al., 1995; Morham et al., 1995). Kidneys from human fetuses exposed to NSAIDs show similar histologic defects (van der Heijden et al., 1994; Morham et al., 1995; Antonucci et al., 2012). These developmental defects may partly be mediated by a reduction in RAS activity. During normal kidney development, changes in COX-2 and renin mRNA levels occur concurrently, related to the fact that COX-2–derived PGE2 stimulates renin release, whereas AT_1_ receptor stimulation subsequently suppresses COX-2. Indeed, COX-2 KO mice do not show the postnatal rise in renin that is essential for normal kidney development (Kaplan et al., 1994). Furthermore, part of the histologic damage in COX-2 KO mice was ameliorated by treatment with an ARB (Kaplan et al., 1994), again illustrating the close COX-PGE2-RAS interaction during kidney development.

The interaction between COX and PGE2 in the kidney medulla is different from its regulation in the kidney cortex and during kidney development. Medullary COX-2 is important for renin release by JG cells. After binding its EP2 or EP4 receptor, PGE2 produced by COX-2 in the thick ascending limb stimulates renin exocytosis from JG cells (Fig. 13) (Schweda et al., 2004). COX-2 expression in the thick ascending limb is regulated by the intratubular chloride concentration, which is sensed by the sodium-potassium-chloride cotransporter 2 (Harris et al., 2004). Dietary salt restriction, diuretics, and ACEIs all increase cortical COX-2 expression and PGE2 production (Harris et al., 1994; Cheng et al., 2002). In isolated perfused tubules, COX-2 inhibition prevented the increase in renin release by low chloride concentrations (Traynor et al., 1999). The increase in renin by dietary salt restriction or ACEIs was also reduced in vivo by COX-2 inhibition and in COX-2 KO mice (Kim et al., 2007). In patients with salt-losing tubulopathies who have high circulating renin levels, cortical COX-2 expression is increased (Kömhoff et al., 2000) and inhibition of COX-2 reduces circulating renin (Blanchard et al., 2015). Similar increases in COX-2 expression have been observed in patients with diabetes and in patients with heart failure (Khan et al., 2001).

Ang II suppresses cortical COX-2 via its type 1 receptor, even at nonpressor doses, and AT_1_ receptor–deficient mice have increased COX-2 expression (Cheng et al., 1999; Zhang et al., 2006). Conversely, activation of the AT_2_ receptor increases cortical COX-2 expression; the net effect of Ang II, however, is a reduction of cortical COX-2 activity (Zhang et al., 2006). As discussed earlier, a link between the (P)RR and COX-2 has also been reported (Kaneshiro et al., 2006; Riquier-Brison et al., 2018). Human (P)RR overexpression increases cortical COX-2 expression in mice, and COX-2 inhibition diminishes kidney blood flow in these animals without a difference in kidney Ang II levels (Kaneshiro et al., 2006). Another study confirmed that (P)RR siRNA and macula densa–specific (P)RR KO reduce COX-2 expression, PGE2 generation, plasma renin, and blood pressure (Riquier-Brison et al., 2018).

In the kidney, medullary COX-2 expression is upregulated by a high-sodium diet (Yang et al., 1998), and PGE2 produced by COX-2 protects against salt-sensitive hypertension by increasing sodium excretion (Zhang et al., 2018) (Fig. 13). This natriuretic effect of COX-2 is stronger in rats with increased RAS activity (Green et al., 2010). In human subjects treated with NSAIDs or COX-2 inhibitors, a transient decrease in sodium excretion with an associated increase in blood pressure or edema is frequently observed (Catella-Lawson et al., 1999). PGE2 production in the kidney medulla is not only increased by high dietary sodium intake but also directly by Ang II. Mice increase medullary COX-2 expression after Ang II infusion (Gonzalez et al., 2014a), and this effect is diminished in mice with CD-specific AT_1_ receptor deletion (Stegbauer et al., 2017). Such KO mice display a stronger increase in blood pressure after Ang II infusion (Stegbauer et al., 2017). Inhibiting either COX-2 or the EP4 receptor also enhances the effect of Ang II infusion on blood pressure (Qi et al., 2002), illustrating the important role of COX-2 and PGE2 in limiting the Ang II–induced sodium retention in the CD. (P)RR activation by prorenin also stimulated COX-2 activity in cortical and in medullary CD cell lines (Gonzalez et al., 2013; Salinas-Parra et al., 2017). ERK1/2 inhibition and short hairpin RNA interfering with (P)RR both prevented this effect, and it did not involve Ang II formation and AT_1_ receptor activation. Moreover, PGE2 increased (pro)renin synthesis in CD cells. The hypothesis is that the upregulation of COX-2 and increased PGE2 production by CD prorenin could counteract some of the effects of Ang II on sodium retention by increasing medullary blood flow and sodium excretion. However, in vivo evidence for this concept is lacking.

### E. Sodium-Glucose Cotransporter-2 Inhibitors

Hyperglycemia induces oxidative stress and inflammation, thus activating the intrarenal RAS (Nishiyama and Kobori, 2018). Subsequently, this renal RAS activation induces hypertension, renal hemodynamic changes, and renal injury, making RAS inhibitors a logical choice to lower blood pressure and slow the progression of kidney injury in patients with DKD. SGLT2 inhibitors were developed as hypoglycemic agents that increase glycosuria by inhibiting reabsorption of filtered glucose within proximal tubules. Yet, recent clinical trials have shown that SGLT2 inhibitors significantly decrease the risk of kidney injury in both diabetic and nondiabetic patients with CKD on top of RAS inhibitors (Zelniker et al., 2019; Cherney et al., 2020). The mechanisms underlying the beneficial effects of SGLT2 inhibitors on kidney outcomes remain unclear. Miyata et al. (2021) recently showed that administration of a SGLT2 inhibitor attenuated glomerular sclerosis and tubulointerstitial fibrosis in Ang II–treated mice independently of changes in blood pressure and GFR. These data suggest a potential role of the intrarenal RAS in the renoprotective effects of SGLT2 inhibitors (Satou et al., 2020).

SGLT2 inhibitors cause reductions in body fluid and blood pressure (Rahman et al., 2017). Thus, it has been proposed that renin release may be activated after administration of SGLT2 inhibitors (Nishiyama and Kobori, 2018). Indeed, several animal studies showed that treatment with SGLT2 inhibitors led to increased plasma renin activity in both diabetic mice (Woods et al., 2019) and nondiabetic CKD rats (Li et al., 2018a), whereas other studies observed no such change (Shin et al., 2016). In patients with type 2 diabetes, SGLT2 inhibitors increased plasma renin, plasma aldosterone, and urinary aldosterone (Eickhoff et al., 2019). Plasma renin also increased in patients with acute heart failure treated with SGLT2 inhibitors (Boorsma et al., 2021). However, plasma aldosterone levels were not affected by SGLT2 inhibitors in nondiabetic healthy volunteers and in patients with acute heart failure (Amin et al., 2015; Boorsma et al., 2021). These contradictory results may be explained by the diuretic properties of SGLT2 inhibitors. Although administration of SGLT2 inhibitors transiently induces diuresis and negatively impacts water balance in the body (Ansary et al., 2017), continuous administration of SGLT2 inhibitors minimizes daily changes in fluid balance (Yasui et al., 2018). Possible effects of SGLT2 inhibitors on sympathetic nerve activity (Wan et al., 2018) may also influence renin release from JG cells.

Treatment with SGLT2 inhibitors significantly lowered urinary AGT levels and renal AGT expression in animal models of type 2 diabetes (Woods et al., 2019), and a similar tendency was observed in patients with type 2 diabetes (Yoshimoto et al., 2017). In contrast, in patients with type 1 diabetes (Cherney et al., 2014) and obese rats fed a high-salt diet (Takeshige et al., 2016), SGLT2 inhibition increased urinary AGT levels. On the other hand, treatment with SGLT2 inhibitors did not alter renal AGT expression in a mouse model of type 1 diabetes (Li et al., 2018a). Thus, the effects of SGLT2 inhibitors on intrarenal AGT remain controversial. However, it is important to note that the intrarenal RAS is likely activated in subjects with diabetes, and one possibility is that the effect of SGLT2 inhibitors relies on the basal activity of the system. Moreover, diabetic and nondiabetic patients with CKD are usually already treated with RAS inhibitors (Heerspink et al., 2020), which are likely to coinfluence the intrarenal RAS.

As discussed in section II.C, the liver is the main source of intrarenal AGT, especially in the setting of proteinuria (Matsusaka et al., 2014). However, AGT is also expressed within proximal tubules and released into urine (Nishiyama and Kobori, 2018). Thus, it is important to unravel the sources of AGT in urine during treatment with SGLT2 inhibitors. Clinical studies have shown that treatment with SGLT2 inhibitors significantly decreases albuminuria in patients with CKD (Heerspink et al., 2020). Consequently, glomerular filtration of circulating AGT may also be reduced by SGLT2 inhibitors, although currently no data support this.

**Fig. 14:**
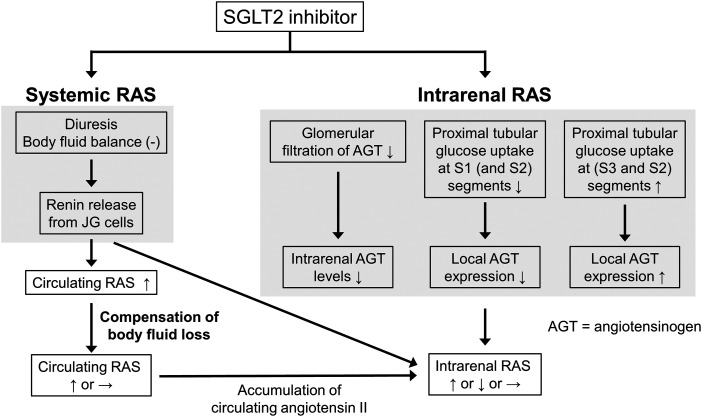
Effects of sodium-glucose cotransporter-2 (SGLT2) inhibitors on the systemic and intrarenal renin-angiotensin system (RAS).

Exposure to high glucose significantly increased AGT mRNA expression in cultured human proximal tubular cells (Wang et al., 2017a). Therefore, a reduction in intracellular glucose levels after treatment with SGLT2 inhibitors may decrease AGT generation in proximal tubular cells. Indeed, treatment with SGLT2 inhibitors or an SGLT2 short hairpin RNA attenuated upregulation of AGT expression after exposure of cultured mouse proximal tubular cells to high glucose (Satou et al., 2020). In diabetic New Zealand obese mice fed a high-fat diet, high levels of AGT expression in renal tissue were decreased after treatment with an SGLT2 inhibitor (Woods et al., 2019). It is important to emphasize, however, that SGLT2 inhibition can increase glucose delivery to the distal proximal tubule (S3 segment), and therefore AGT production at this site may be stimulated. Thus, different effects of SGLT2 inhibitors on AGT production at early and late proximal tubules may lead to inconsistent conclusions regarding responses of intrarenal RAS to SGLT2 inhibition. Indeed, some studies have found that SGLT2 inhibitors lower renal tissue AGT expression (Han et al., 2018), whereas others have shown the opposite (Wang et al., 2017b) using the same diabetes mouse model. The potential mechanisms through which SGLT2 inhibitors may influence the intrarenal RAS are illustrated in Fig. 14.

## VI. Conclusions

Local angiotensin formation in the kidney is a rapidly evolving field, which started many years ago as an ‘independent system’ relying on AGT synthesis in the proximal straight tubule and prorenin synthesis in the CD. The latter would act on/via the (P)RR, and urinary AGT and prorenin would then reflect renal RAS activity. In reality, we now know that angiotensin generation at renal tissue sites fully depends on AGT of hepatic origin, whereas CD (pro)renin rather reflects uptake of filtered urinary (pro)renin that has not yet been reabsorbed. Megalin turns out to be a novel player, reabsorbing both AGT and (pro)renin, and consequently, the urinary levels of these RAS components largely reflect the net effect of glomerular filtration and megalin-mediated tubular reabsorption. Since both filtration and megalin expression are altered in disease conditions (like DKD), this will directly affect the urinary levels of AGT, renin, and prorenin, thus explaining why their levels correlate with disease severity. In fact, megalin-dependent AGT reabsorption may underlie renal angiotensin generation, particularly after podocyte injury, which will greatly increase glomerular leakage of plasma AGT. Under healthy conditions, renal Ang II is probably generated from liver-derived AGT in the capillary lumen or interstitium since filtration/reabsorption then does not yield sufficient AGT. Given that renal angiotensin generation depends on hepatic AGT, it is not surprising that liver-targeted AGT siRNA and antisense oligonucleotides effectively suppress the renal RAS and may thus exert renoprotection in a blood pressure–independent manner.

Megalin-mediated reabsorption depends on the (P)RR, but this receptor as well as its soluble variant s(P)RR now turn out to have a wide variety of effects on kidney function (water transport, sodium reabsorption) and fibrosis that are unrelated to the RAS. Hence, (P)RR-(pro)renin interaction underlying multiple renal effects currently is a less likely scenario. Nevertheless, this implies that interfering with the (P)RR might offer beneficial effects on top of RAS blockade. The major effector of the RAS remains the AT_1_ receptor, with possible modulatory (counteracting) roles for both the AT_2_ receptor and Mas receptor. Here an urgent question is what agonist activates these latter two receptors since generally the preferred agonists of the AT_2_ receptor (Ang III) and the Mas receptor [Ang-(1-7)] occur at much lower levels than those of Ang II, if detectable at all. This may differ under conditions where RAS blockers are introduced or after the application of soluble ACE2, which converts Ang II to Ang-(1-7). Since membrane-bound ACE2 is simultaneously the main cell entry receptor for SARS-CoV-2, its application as a soluble variant may function as a decoy, preventing cellular accumulation of the virus. Kidney organoids would allow us not only to study this concept in a human in vitro setting but also to explore novel and existing drugs. ARNI and SGLT2 inhibitors have now entered the clinic and are currently being studied closely because of their renoprotective effects. This may involve interference with the renal RAS, although neprilysin inhibition alone does not appear to alter renal angiotensin generation. Finally, COX inhibition may reemerge as a renoprotective approach in hyperfiltrating and albuminuric CKD patients by reducing glomerular perfusion pressure, with similar short-term benefits as RAS inhibition. However, given the systemic side effects of COX inhibition, a more likely scenario is interfering at the level of one or more of the prostaglandin receptors, in particular those of PGE2, which are linked to both renin synthesis and the (P)RR. Taken together, local synthesis of renin and prorenin at the site of the JG apparatus, with the capacity of an upregulation of several 100-fold by transforming VSMCs along the renal arterioles into renin cells, combined with megalin-mediated uptake of hepatic AGT and the occurrence of a wide variety of angiotensinases and AT receptors, allow the kidney to possess a local RAS that acts independently of the circulating RAS. A wide variety of existing and novel drugs exert beneficial renal effects by interfering with this intrarenal RAS.

## Abbreviations

ACE: angiotensin-converting enzyme
ACEI: ACE inhibitor
ADAM: a disintegrin and metalloprotease
ADPKD: autosomal dominant polycystic kidney disease
AGT: angiotensinogen
Ang: angiotensin
APA: aminopeptidase A
ARB: AT_1_ receptor blocker
ARNI: angiotensin receptor/neprilysin inhibition
AT_1_: Ang II type 1
AT_2_: Ang II type 2
ATPase: adenosine triphosphatase
AT_1_R: angiotensin II type 1 receptor
AT_2_R: angiotensin II type 2 receptor
C21: compound 21
CD: collecting duct
CKD: chronic kidney disease
COVID-19: coronavirus disease 2019
COX: cyclooxygenase
CREB: cAMP-responsive element-binding protein
DDP: dipeptidyl aminopeptidase III
DKD: diabetic kidney disease
eGFR: estimated glomerular filtration rate
ENaC: epithelial Na^+^ channel
EP: specific PGE2 receptor
ERK1/2: extracellular signal-regulated kinase 1/2
Foxd1: forkhead box protein D1
GFR: glomerular filtration rate
HCO_3_^−^: bicarbonate
IM: inner medulla
JG: juxtaglomerular
KO: knockout
L-NAME: N^G^-nitro-L-arginine methyl ester
MAPK: mitogen-activated protein kinase
NEP: neutral endopeptidase/neprilysin
NF-*κ*B: nuclear factor-kappa B
NHE3: Na^+^/H^+^ exchanger 3
NO: nitric oxide
NSAID: nonsteroidal anti-inflammatory drug
PGE2: prostaglandin E2
PGI2: prostaglandin I2
POP: prolyloligopeptidase
PRCP: prolylcarboxypeptidase
(P)RR: (pro)renin receptor
RAS: renin-angiotensin system
RMIC: renomedullary interstitial cell
SARS-CoV-2: severe acute respiratory syndrome coronavirus 2
SGLT2: sodium-glucose cotransporter-2
SHR: spontaneously hypertensive rat
siRNA: small interfering RNA
s(P)RR: soluble (pro)renin receptor
STZ: streptozotocin
TGF*β*1: transforming growth factor *β*1
VSMC: vascular smooth muscle cell.
